# Tunable nonenzymatic degradability of *N*-substituted polyaspartamide main chain by amine protonation and alkyl spacer length in side chains for enhanced messenger RNA transfection efficiency

**DOI:** 10.1080/14686996.2019.1569818

**Published:** 2019-02-13

**Authors:** Mitsuru Naito, Yuta Otsu, Rimpei Kamegawa, Kotaro Hayashi, Satoshi Uchida, Hyun Jin Kim, Kanjiro Miyata

**Affiliations:** a Center for Disease Biology and Integrative Medicine, Graduate School of Medicine, The University of Tokyo, Tokyo, Japan; b Department of Materials Engineering, Graduate School of Engineering, The University of Tokyo, Tokyo, Japan; c Innovation Center of NanoMedicine, Kawasaki Institute of Industrial Promotion, Kawasaki, Japan; d Department of Bioengineering, Graduate School of Engineering, The University of Tokyo, Tokyo, Japan

**Keywords:** Degradability, polycation, poly(amino acid), polyion complex, mRNA, 30 Bio-inspired and biomedical materials, 211 Scaffold / Tissue engineering / Drug delivery

## Abstract

Degradability of polycations under physiological conditions is an attractive feature for their use in biomedical applications, such as the delivery of nucleic acids. This study aims to design polycations with tunable nonenzymatic degradability. A series of cationic *N*-substituted polyaspartamides were prepared to possess primary amine via various lengths of alkyl spacers in side chains. The degradation rate of each polyaspartamide derivative was determined by size exclusion chromatography under different pH conditions. The *N*-substituted polyaspartamide containing a 2-aminoethyl moiety in the side chain (PAsp(AE)) showed considerable degradability under physiological conditions (pH 7.4, 37 °C). In contrast, the *N*-substituted polyaspartamides bearing a longer alkyl spacer in the side chain, i.e. the 3-aminopropyl (PAsp(AP)) and 4-aminobutyl moieties (PAsp(AB)), more strongly suppressed degradation. Further, a positive correlation was observed between the degradation rate of *N*-substituted polyaspartamides and a deprotonation degree of primary amines in their side chains. Therefore, we conclude that the deprotonated primary amine in the side chain of *N*-substituted polyaspartamides can induce the degradation of the main chain through the activation of amide nitrogen in the side chain. When *N*-substituted polyaspartamides were utilized as a messenger RNA (mRNA) delivery vehicle via formation of polyion complexes (PICs), degradable PAsp(AE) elicited significantly higher mRNA expression efficiency in cultured cells compared to PAsp(AP) and PAsp(AB). The higher efficiency of PAsp(AE) might be due to the facilitated destabilization of PICs within the cells, directed toward mRNA release. Additionally, degradation of PAsp(AE) considerably reduced its cytotoxicity. Thus, our study highlights a useful design of well-defined cationic poly(amino acid)s with tunable nonenzymatic degradability.

## Graphical abstract

**Figure:**
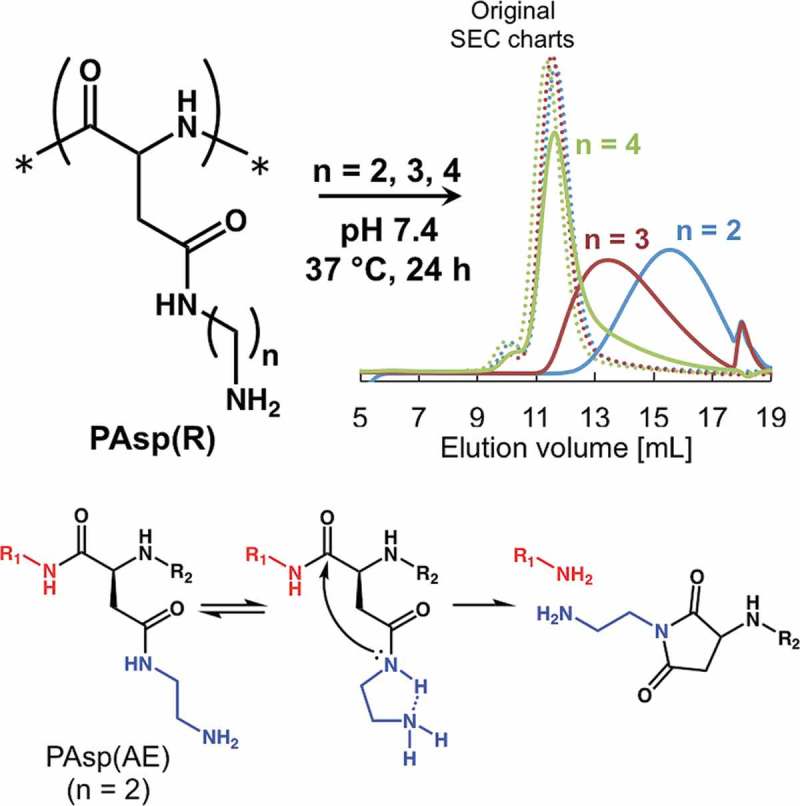

## 1. Introduction

Biodegradable polymers have been widely used in biomaterial applications, such as drug delivery [1,2] and tissue engineering [3,4]. The biodegradability of polymers is a great advantage because degraded products or fragments are more rapidly metabolized or eliminated from the body, compared to the parent polymers. Biodegradable polymers are likely to elicit low cytotoxicity compared to the nondegradable polymers [5,6]. The drug delivery systems fabricated using biodegradable polymers allow for time-dependent (or controlled) release of drug payloads [7]. The drug releasability is particularly important for nucleic acid delivery [8]. Vulnerable nucleic acids, such as small interfering RNA (siRNA) and messenger RNA (mRNA), need delivery vehicles for protection against nuclease degradation. Polyion complexes (PICs) fabricated between the nucleic acid and the polycations have been extensively developed to fulfill the demand for delivery of nucleic acids [9–12]. Previous studies have demonstrated that polycations with a high positive charge are desirable for fabricating stable PICs with nucleic acids through electrostatic interactions [13,14]. However, such stable PICs in turn may compromise the release efficiency of nucleic acid payload in the target cells. Thus, the availability of novel biodegradable polycations with efficient payload releasability and low cytotoxicity still remains a major challenge in nucleic acid delivery.

**Scheme 1.**
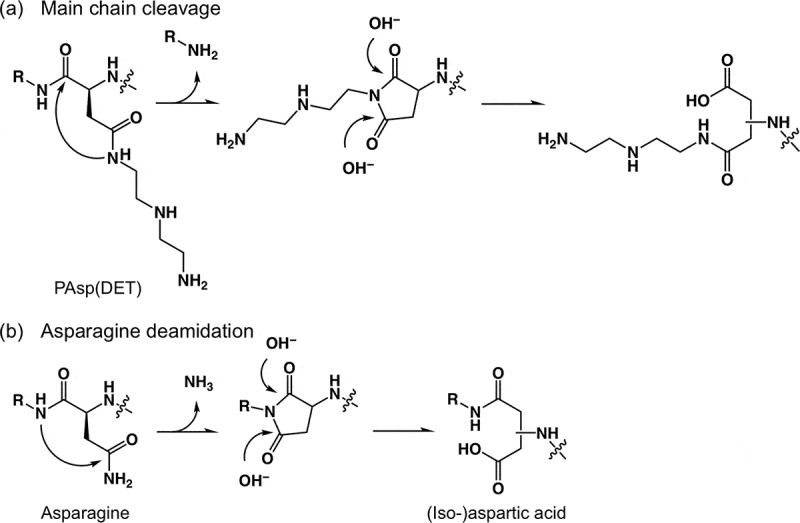
Mechanisms of (a) main chain cleavage of PAsp(DET) and (b) asparagine deamidation.

Most of the degradable polymers contain ester bonds or other biodegradable linkers, such as the disulfide bond and acetal bond. These chemical bonds often restrict the polymer design and/or the handling method. With regard to biodegradable polycations, Schmitt et al. [15] reported that *N*-substituted polyaspartamides bearing a 2-aminoethyl (-CH_2_CH_2_NH_2_) moiety in the side chain were nonenzymatically degraded in aqueous milieu, and that their degradation rates increased under basic conditions. We previously developed *N*-substituted polyaspartamide bearing a two-repeated 2-aminoethyl moiety (-(CH_2_CH_2_NH)_2_-H) in the side chains (PAsp(DET)), which elicited nonenzymatic degradation associated with minimal cytotoxicity, and thus enabled the highly efficient transfection of plasmid DNA (pDNA) [16–18], mRNA [19,20], and oligonucleotide/protein complex [21]. Interestingly, the degradability of PAsp(DET) arose from a cleavage of the amide bond in the main chain, which tends to be a stable bond in the absence of degradation enzymes in physiological conditions. It was postulated that the cleavage of the main chain of PAsp(DET) should be ascribed to the nucleophilic attack of the amide nitrogen in the side chain (CO***N***H_side_) at the amide carbon in the main chain (***C***ONH_main_) (Scheme 1(a)) [17]. An analogous reaction, asparagine (or glutamine) deamidation, occurs in the body through the nucleophilic attack of CO***N***H_main_ on ***C***ONH_side_ accompanied by elimination of ammonia from the carbamoyl group in the side chain, resulting in peptide isomerization (Scheme 1(b)) [22,23]. The main chain cleavage in PAsp(DET) is therefore, an unnatural reaction, as the CO***N***H_side_ activation process remains to be elucidated.

**Scheme 2.**
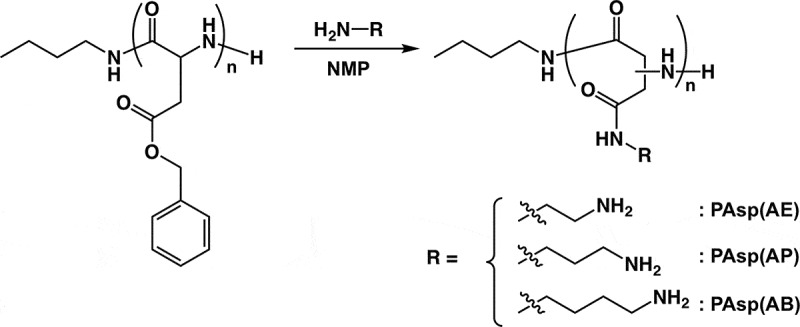
Synthesis of PAsp(AE), PAsp(AP), and PAsp(AB) by aminolysis of PBLA with corresponding diamino compounds.

Herein, we hypothesize that the side-chain amino groups in PAsp(DET) would activate the nearby CO***N***H_side_ through proton abstraction. To confirm our hypothesis, a series of *N*-substituted polyaspartamides containing primary amines with varying lengths of alkyl spacers in the side chains, i.e. 2-aminoethyl moiety (PAsp(AE)), 3-aminopropyl moiety (PAsp(AP)), and 4-aminobutyl moiety (PAsp(AB)), were synthesized from the same parent polymer with a narrow molecular weight distribution (Scheme 2). The *N*-substituted polyaspartamides obtained were characterized for the degradability under physiological conditions to confirm the effects of the length of the alkyl spacer and the level of protonation of the primary amines in the side chain. The *N*-substituted polyaspartamides were added to cultured cells to evaluate the effect of degradability on cytotoxicity. Finally, mRNA-loaded PICs were prepared from each of the *N*-substituted polyaspartamides to demonstrate that the degradability of polycations led to significantly enhanced mRNA transfection efficiency.

## 2. Materials and methods

### 2.1. Materials

β-Benzyl-l-aspartate *N*-carboxyanhydride (BLA-NCA) was purchased from Chuo Kaseihin Co. Inc. (Tokyo, Japan). *n*-Butylamine and 1,3-diaminopropane (DAP) were purchased from Tokyo Chemical Industry Co. Ltd. (Tokyo, Japan). *N*-Methyl-2-pyrrolidone (NMP), benzene, *N,N*-dimethylformamide (DMF), dichloromethane (CH_2_Cl_2_), thiourea, lithium bromide (LiBr), dimethyl sulfoxide-*d6* (containing 0.05 vol% tetramethylsilane), ethylenediamine (EDA), 1,4-diaminobutane (DAB), sodium chloride (NaCl), acetic acid, deuterium oxide, sodium 3-(trimethylsilyl) propionate-2,2,3,3-*d*_4_, glycine, sodium dihydrogen phosphate dihydrate, disodium hydrogen phosphate 12-water, Dulbecco’s modified Eagle’s medium (DMEM), fetal bovine serum, and Dulbecco’s phosphate-buffered saline (D-PBS) were purchased from FUJIFILM Wako Pure Chemical Corporation (Osaka, Japan). 2-[4-(2-Hydroxyethyl)-1-piperazinyl]ethanesulfonic acid (HEPES) was purchased from Dojindo Laboratories (Kumamoto, Japan). Trypsin-ethylenediamine tetraacetate and penicillin-streptomycin (P/S) were purchased from Sigma-Aldrich Co. (St. Louis, MO, USA). EDA, DAP, DAB, NMP, and DMF were distilled with calcium hydride (CaH_2_) under reduced pressure. CH_2_Cl_2_ was distilled with CaH_2_ under normal pressure. A human hepatoma cell line (Huh-7) was obtained from the Japanese Collection of Research Bioresources Cell Bank (Osaka, Japan). Huh-7 cells were cultured in DMEM with 10% FBS (v/v) containing 1% P/S and incubated in 5% CO_2_ at 37 °C.

### 2.2. Synthesis of poly(β-benzyl-*l*-aspartate) (PBLA)

PBLA was synthesized via ring-opening polymerization of BLA-NCA initiated by the primary amino group of *n*-butylamine as reported previously [14]. Briefly, BLA-NCA (1.35 g, 5.42 mmol) was mixed with *n*-butylamine (4.87 μL, 49.3 μmol) as an initiator in the solution containing a mixture of distilled CH_2_Cl_2_ and distilled DMF. This solution was continuously stirred at 35 °C for 3 days. The completion of polymerization was confirmed based on the disappearance of peaks in the infrared (IR) spectrum corresponding to the carboxylic anhydride group of NCA in an IR Report-100 instrument (JASCO Corporation, Tokyo, Japan). The reaction mixture was poured into a large amount of diethyl ether to precipitate PBLA, and was followed by vacuum-drying. The molecular weight distribution (*M*_w_/*M*_n_) of PBLA was analyzed by size exclusion chromatography (SEC) (GPC system, Shimadzu Corporation, Kyoto, Japan) using a TSKgel column (SuperAW4000). NMP with 50 mM LiBr was used as an eluent (Figure S1(a)). The degree of polymerization (DP) of PBLA was determined based on the peak intensity ratio of methyl protons in the initiator (C*H*_3_CH_2_–, *δ* = 0.8) to benzyl protons (C_6_ *H*_5_CH_2_–, *δ* = 7.3) in the proton nuclear magnetic resonance (^1^H NMR) spectrum (JNM-ECS 400, JEOL Resonance Inc., Tokyo, Japan) (Figure S1(b)).

### 2.3. *Synthesis of* N*-substituted polyaspartamide, PAsp(R)*

A series of PAsp(R)s were synthesized via aminolysis of benzyl groups in PBLA with varying diamines as reported previously, but with minor modifications [14]. Typically, PBLA (100 mg, 4.60 μmol) was lyophilized in benzene and dissolved in distilled NMP containing 0.5 M of thiourea (20 mg/mL) at 35 °C. Separately, each diamino compound (50 equivalent to BLA unit) was mixed with the same volume of distilled NMP containing 0.5 M thiourea. Both solutions were cooled to 4 °C, after which, the PBLA solution was added dropwise to the diamino compound solution. The reaction solution was stirred at 4 °C for 1 h, and neutralized with hydrochloric acid at a temperature below 10 °C. This was followed by overnight dialysis with 0.01 M HCl first and then with deionized water for 2 h at 4 °C. The dialyzed solutions were lyophilized to obtain a series of PAsp(R)s. The *M*_w_/*M*_n_ value of each PAsp(R) was analyzed by SEC (eluent: 10 mM acetic acid with 500 mM NaCl) equipped with UV-1575 (JASCO Corporation) and superdex 75 10/300 GL (GE Healthcare UK Ltd., UK) as the separation column (Figure S2(a)). Quantitative aminolysis was confirmed based on the peak intensity ratio of the PAsp(R) side-chain protons (-CONH(C*H*_2_)_2_NH_2_, *δ* = 3.2 and 3.6 for PAsp(AE), -CONH(C*H*_2_)_3_NH_2_, *δ* = 1.9, 3.1, and 3.3 for PAsp(AP), and -CONH(C*H*_2_)_4_NH_2_, *δ* = 1.6–1.8, 3.1, and 3.3 for PAsp(AB)) to the main-chain protons (CHC*H*_2_CONH, *δ* = 2.6–3.0) in the ^1^H NMR spectra (Figure S2(b–d)).

### 2.4. Potentiometric titration

A series of PAsp(R)s were dissolved at a concentration of 10 mg/mL in 0.1 M HCl containing 150 mM NaCl. These solutions were titrated at 37 °C with 0.1 M NaOH containing 50 mM NaCl as a titrant with an automatic titration device, TS-2000 (Hiranuma Sangyo Co., Kyoto, Japan). The degree of protonation for each PAsp(R) was calculated from the titration curve obtained (Figure S3).

### 2.5. *Measurement of degradation of* N*-substituted polyaspartamides*

A series of PAsp(R)s were dissolved at a concentration of 1 mg/mL in 100 mM acetate buffer with 110 mM NaCl (pH 5.0), 80 mM phosphate buffer (pH 7.4), 100 mM HEPES buffer with 80 mM NaCl (pH 8.0), 100 mM HEPES with 90 mM NaCl (pH 8.5), 100 mM glycine with 120 mM NaCl (pH 9.0), and 100 mM glycine with 110 mM NaCl (pH 10.0). The pH of the buffers was adjusted with NaOH. The ionic strength of each buffer was adjusted to resemble the physiological condition (*I* = 0.16). The solution was maintained at 37 °C with gentle mixing for scheduled periods. Each PAsp(R) solution was analyzed by SEC (eluent: 10 mM acetic acid with 500 mM NaCl) equipped with UV-1575 (JASCO corporation), and superdex 75 10/300 GL (GE Healthcare UK Ltd) as the separation column. The number-average molecular weight (*M*_n_) of each sample was calculated using the ChromNAV software (JASCO corporation).

### 2.6. *Determination of the cleavage rate constant of* N*-substituted polyaspartamides*

The cleavage of amide bonds in the *N*-substituted polyaspartamide main chain is a unimolecular reaction. So, the cleavage rate is expressed as a first-order reaction using the number of residual amide bonds in the main chain (*n*_b_) at time *t*, as follows [24].

(1) $$-dn_{b}/dt=kn_{b}$$

where, *k* [h^–1^] is the cleavage rate constant.

Equation (1) can be transformed and simplified as below:

(2) $$f=exp\left(-kt\right)$$

where *f* is the fraction of residual amide bonds in the main chain per polymer.

Equation (2) can be further transformed as below:

(3) lnf= −kt.

Thus, the cleavage rate constant *k* can be obtained from the slope by plotting ln*f* against *t*. Here, *f* is expressed using the number of initial amide bonds (*n*_b,0_) and the cleaved amide bonds (*n*_c_) as below:

(4) $$f=\;\left(n_{b,0}-n_{c}\right)/n_{b,0}.$$

Also, *M*_n_ at time *t* is expressed using *n*_c_ and the initial *M*_n_ at *t* = 0 (*M*_n,0_) as below:

(5) $$M_{n}/M_{n,0}=1/\left(n_{c}+\;1\right).$$

Equations (4) and (5) yield:

(6) $$f=\;\left(1\;+n_{b,0}-M_{n,0}/M_{n}\right)/n_{b,0}.$$

Thus, the measurement of *M*_n_ value yields *f*.

### 2.7. *Cytotoxicity of* N*-substituted polyaspartamides*

Huh-7 cells were seeded in a 96-well plate (5000 cells/well) and incubated for 24 h. Each of the polyaspartamide solution was added to the cultured cells at desired concentrations and incubated for 48 h. Cell viability was determined using the Cell counting kit-8 (Dojindo Laboratories) and a microplate reader (Infinite M1000 PRO, Tecan group Ltd., Zürich, Switzerland), following the manufacturer’s protocol.

### 2.8. mRNA preparation

*Gaussia luciferase* (*GLuc*) mRNA was prepared as reported previously [25]. Briefly, the template pDNA (pT7-GLuc-pA) was prepared by inserting *GLuc* sequence from the pCMV-GLuc vector (New England BioLabs, Ipswich, MA, USA) and a 120 bp poly A/T sequence into the pSP73 vector construct (Promega Corporation, Madison, WI, USA). After linearization of the pT7-GLuc-pA DNA to obtain the template DNA, the template DNA was transcribed *in vitro* using the mMESSAGE mMACHINE T7 ULTRA Kit (Life Technologies, Carlsbad, CA, USA) following the manufacturer’s protocol. The mRNA obtained was purified using the RNeasy mini kit (Qiagen, Hilden, Germany). The high purity of the mRNA was confirmed using an Agilent 2100 Bioanalyzer in combination with the Agilent RNA 6000 Nano Kit (Agilent, Santa Clara, CA, USA).

### 2.9. Preparation of mRNA-loaded PICs

A series of PAsp(R)s were separately dissolved in 10 mM HEPES buffer (pH 7.4) at a concentration of 0.1 mg/mL. Each solution was mixed with mRNA (100 ng/μL in 10 mM HEPES buffer) such that the residual molar ratio of primary amines in PAsp(R) to phosphates in mRNA is 5. The scattered light intensities (SLIs), hydrodynamic diameters, and polydispersity indices (PDIs) of mRNA-loaded PICs were measured using a Zetasizer Nano ZS (Malvern Instruments Ltd., UK) equipped with a He–Ne laser (633 nm incident beam).

### 2.10. Transfection of mRNA-loaded PICs

Huh-7 cells were seeded in a 96-well plate (5000 cells/well) and incubated for 24 h. The *GLuc* mRNA-loaded PICs were added to each well (250 ng of mRNA/well). The *GLuc* expression level was determined using the supernatant (10 µL) of the culture medium from each well 24 h after transfection. The photoluminescence intensity was determined based on the Renilla luciferase assay system (Promega Corporation) and Mithras LB940 luminometer (Berthold Technologies, Bad Wildbad, Germany).

### 2.11. Flow cytometry

*GLuc* mRNA was labeled with Cy5 using a Label IT Cy5 Labeling Kit (Mirus Bio, Madison, WI, USA). Huh-7 cells were seeded in a 6-well plate (100,000 cells/well) and incubated for 24 h. To each well, Cy5-labeled mRNA (Cy5-mRNA)-loaded PICs were added (250 ng of mRNA/well). After 24 h incubation, the transfected cells were washed twice with cold PBS and collected after trypsinization. The cells were centrifuged and resuspended in PBS. The Cy5 intensity of the cells treated with Cy5-mRNA was measured in a BD LSR II flow cytometer (BD Biosciences, Franklin Lakes, NJ, USA), equipped with a 633 nm laser and 660/20 nm filter.

## 3. Results and discussion

### 3.1. *Synthesis of* N*-substituted polyaspartamides*

A precursor polymer, PBLA, with a narrow molecular weight distribution (*M*_w_/*M*_n_ = 1.13, DP = 106) was synthesized (Figure S1). The completion of NCA polymerization was confirmed by the disappearance of NCA-BLA signal in the IR spectrum (1860 and 1780 cm^–1^ for carboxylic anhydride) (data not shown). PBLA was subjected to aminolysis to obtain a series of amine-functionalized PAsp(R)s with varying alkyl spacers in the side chains. DAE, DAP, and DAB reacted with PBLA to synthesize PAsp(R) comprising the 2-aminoethyl group (PAsp(AE)), 3-aminopropyl group (PAsp(AP)), and 4-aminobutyl group (PAsp(AB)), respectively, in the side chains (Scheme 2). The quantitative conversions of PBLA to PAsp(R)s were confirmed by ^1^H NMR (Figure S2(b–d)). Note that the *M*_w_/*M*_n_ values were not altered before and after aminolysis (*M*_w_/*M*_n_ < 1.2) (Figure S2(a)).

### 3.2. *Degradation measurements of* N*-substituted polyaspartamides*

**Figure 1.**
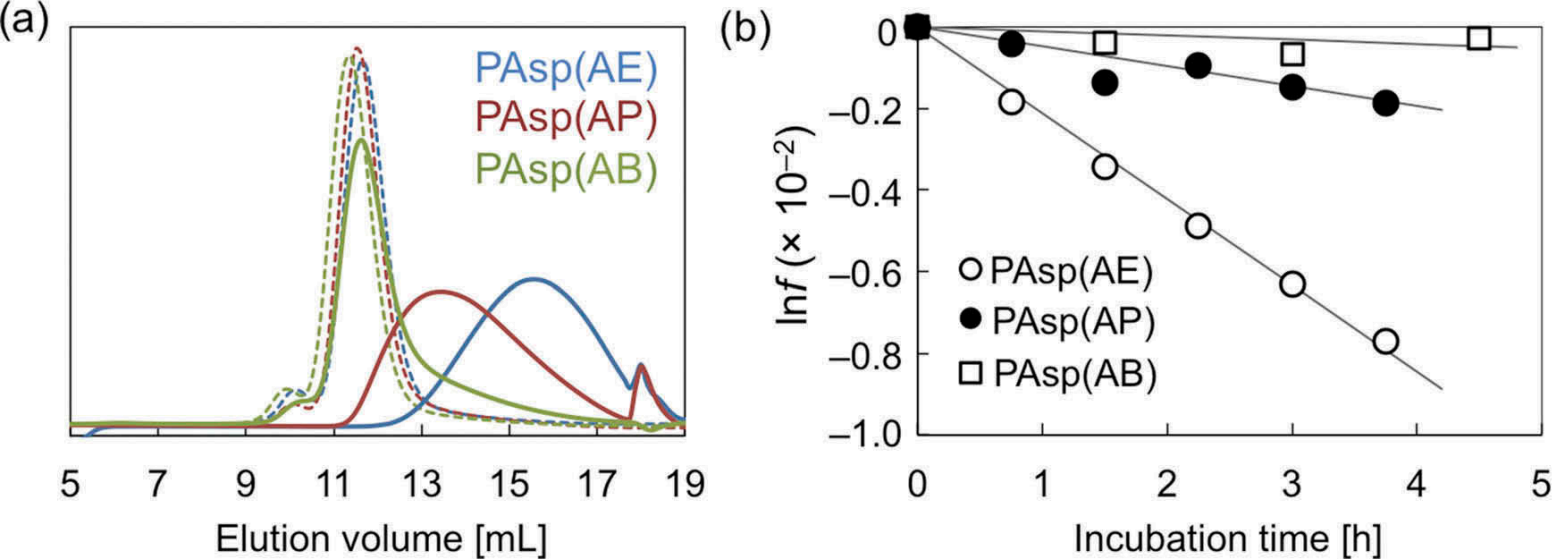
(a) SEC chart showing PAsp(AE), PAsp(AP), and PAsp(AB) before (dotted line) and after incubation for 24 h (solid line) under the physiological condition (pH 7.4, 37 °C); (b) ln*f* of PAsp(AE) (○), PAsp(AP) (●), and PAsp(AB) (□) plotted against incubation time.

The degradability of each PAsp(R) was evaluated by SEC analysis in physiological conditions (pH 7.4, 37 °C). The chromatogram obtained for PAsp(AE) for the 24 h incubation revealed the generation of low molecular weight fragments at around 13–19 mL in the elution volume (Figure 1(a)), demonstrating the nonenzymatic degradability of PAsp(AE). To clarify which of the amide bonds in the main or the side chains were cleaved, the PAsp(AE) sample was further incubated for 72 h under the physiological condition and was analyzed by SEC and ^1^H NMR. The SEC chart reveals that after the 72 h incubation, PAsp(AE) generated even lower molecular weight fragments (15–19 mL in elution volume) (Figure S4(a)). This indicates that the degradation reaction was further progressed. Also, the ^1^H NMR spectrum displays only a slight peak derived from free EDA (*δ* = 3.4) for the PAsp(AE) sample even after extended incubation for 72 h (Figure S4(b)); the intensity ratio of this peak to those from the AE moiety (δ = 3.2 and 3.6) was calculated to be ~6%. These results indicate that few cleavage reactions occurred in the side-chain amide bonds of PAsp(AE). Therefore, we conclude that the degradability of PAsp(AE) under the physiological condition (pH 7.4, 37 °C) was mostly due to the cleavage of the amide bond in the main chain.

The peak observed in the SEC charts was progressively shifted toward smaller elution volume with an increase in the alkyl spacer length of PAsp(R) side chains (Figure 1(a)), indicating the considerable effect of the length of alkyl spacers on the degradability of *N*-substituted polyaspartamides. To quantitatively evaluate their degradability, each PAsp(R) was incubated for shorter periods of time from 45 to 270 min under the same physiological conditions (pH 7.4, 37 °C) and was analyzed by SEC (Figure S5). The relative *M*_n_ (= *M*_n_/*M*_n,0_) values were calculated and plotted against incubation time (Figure S6). Relative *M*_n_ values of PAsp(AE) decreased more rapidly compared to the other polyaspartamides. Assuming that the main-chain amide bond cleavage is a unimolecular reaction, the cleavage rate constant (*k* [h^–1^]) in the cleavage reaction (Scheme S1) was obtained according to Equation (3) by plotting the ln*f* values against incubation time (Figure 1(b)), as summarized in Table 1. The linear correlation was clearly observed between ln*f* and *t*, confirming that the cleavage reaction occurred at a constant rate during degradation. This indicates that the degradation process of PAsp(R)s was independent from the degraded states (or DP). It should be noted that the *k* value of PAsp(AE) at pH 7.4 (2.11 × 10^–3^ [h^–1^]) was much higher than that for a hydrolysis reaction of poly(l-lactide) at pH 7.4 and 37 °C (1.08 × 10^–4^ [h^–1^]) [26]. From the obtained *k* values, the incubation time at which the relative *M*_n_ reaches 0.5 (*T* [h]) was calculated as 4.5, 19.8, and 91.2 for PAsp(AE), PAsp(AP), and PAsp(AB), respectively (Table 1). Thus, the degradability of PAsp(AE) at pH 7.4 was estimated to be 20 times faster than that of PAsp(AB), and 4 times faster than that of PAsp(AP).

**Table 1.** Cleavage rate constant (*k*) [h^–1^] and *T* [h] of PAsp(R)s.

Polymer		pH 5.0	pH 6.5	pH 7.4	pH 8.0	pH 8.5	pH 9.0	pH 10.0
PAsp(AE)	*k*	1.86 × 10^–5^	8.36 × 10^–4^	2.11 × 10^–3^	1.08 × 10^–2^	1.61 × 10^–2^	2.44 × 10^–2^	–
	*T*	514	11.5	4.5	0.89	0.59	0.39	–
PAsp(AP)	*k*	–	–	4.84 × 10^–4^	–	–	9.38 × 10^–4^	1.76 × 10^–3^
	*T*	–	–	19.8	–	–	10.2	5.4
PAsp(AB)	*k*	–	–	1.05 × 10^–4^	–	–	2.25 × 10^–4^	4.50 × 10^–4^
	*T*	–	–	91.2	–	–	42.6	21.3

**Figure 2.**
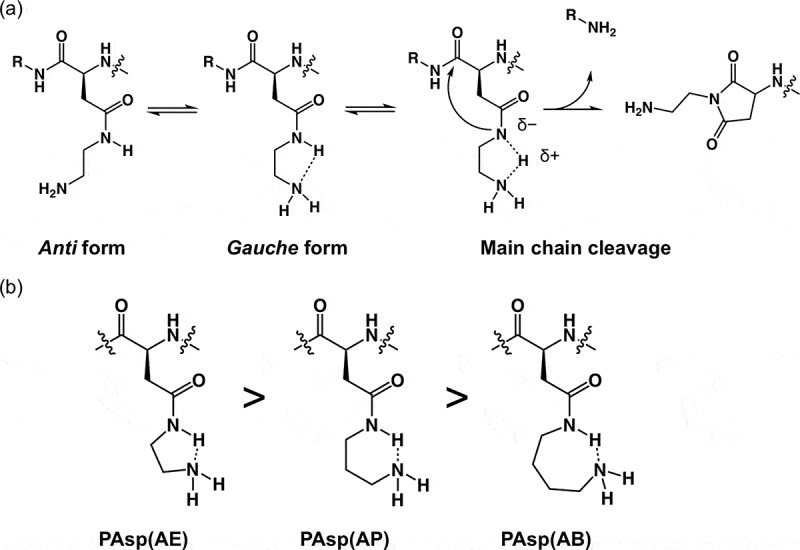
(a) Expected conformational changes in the side chain of PAsp(AE); and (b) expected ring-like conformations in the side chain of PAsp(R) series and the stabilization tendency are shown.

The varying degradation profiles of PAsp(R)s reveal that the alkyl spacer length between the amide bond and the primary amine in the side chains significantly affect the degradability of *N*-substituted polyaspartamides. Particularly, the ethylene spacer in PAsp(AE) enabled a very rapid degradation, which can be explained by the five-membered ring-like conformation in the side chain for activation of CO***N***H_side_ (or abstraction of amide proton), as illustrated in Figure 2(a). Through this, the activated CO***N***H_side_ might initiate the cleavage of the amide bond in the main chain via nucleophilic attack of CO***N***H_side_ on ***C***ONH_main_. The six- and seven-membered ring-like conformations in PAsp(AP) and PAsp(AB), respectively, may be energetically unfavorable compared to the five-membered ring-like conformation in PAsp(AE) (Figure 2(b)) [27], and consistent with the much slower degradation profiles of PAsp(AP) and PAsp(AB).

### 3.3. *pH-dependent degradation measurements of* N*-substituted polyaspartamides*

**Figure 3.**
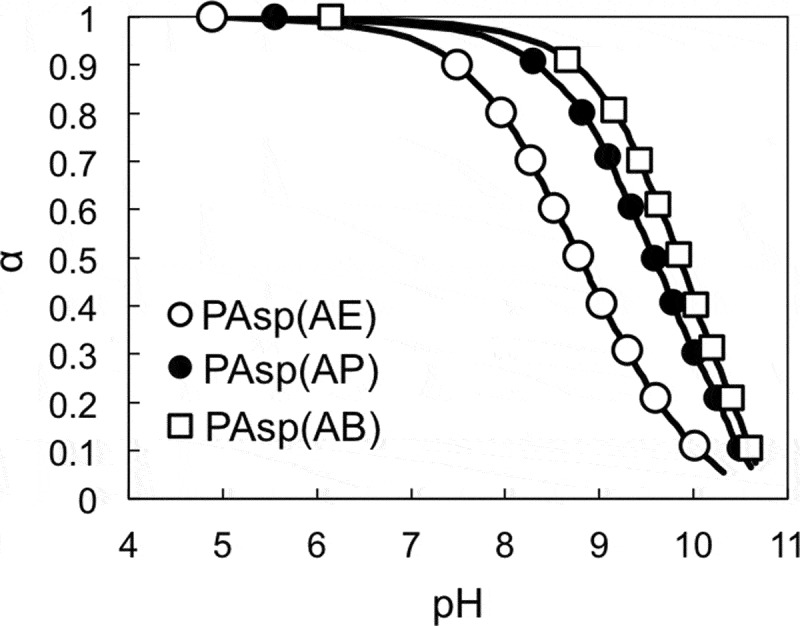
α/pH curves of PAsp(AE) (○), PAsp(AP) (●), and PAsp(AB) (□).

To validate the aforementioned hypothesis that the primary amine in the side chain of PAsp(R)s might activate CO***N***H_side_ toward main chain cleavage, the degradability of PAsp(R)s was further examined by focusing on the protonation state of the primary amines. Considering that the protonated primary amines with low nucleophilicity cannot abstract the amide proton (Figure S7), the degradation rate of the PAsp(R) series should be modulated according to the protonation degree (α) of primary amines. Prior to the degradation measurements, each PAsp(R) was subjected to potentiometric titration to determine the α of the primary amines. The obtained α/pH curves show PAsp(R) with longer alkyl side chains possessing higher p*K*_a_ values, i.e. 8.8, 9.6, and 9.9 for PAsp(AE), PAsp(AP), and PAsp(AB), respectively (Figure 3). This shift in p*K*_a_ could be explained by the difference in the electrostatic repulsion between the protonated primary amines. The longer the alkyl spacer, the more able it is to mitigate electrostatic repulsion and accelerate the protonation of primary amines.

**Figure 4.**
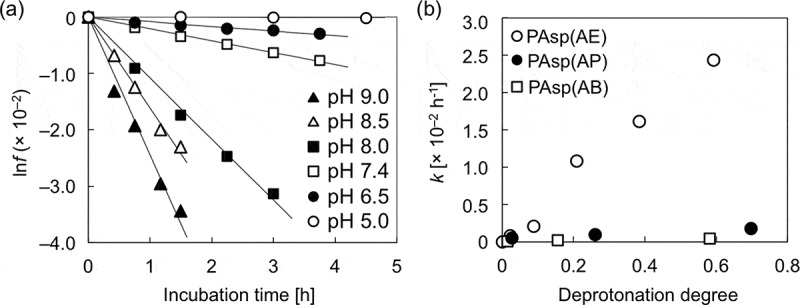
(a) ln*f* of PAsp(AE) at different pH values; (b) changes in cleavage rate constant (*k*) as a function of the degree of deprotonation of the primary amines in the side chains of PAsp(AE) (○), PAsp(AP) (●), and PAsp(AB) (□).

The degradation behavior of PAsp(AE) after incubation for 45 min was affected by pH, as shown in the SEC chart (Figure S8). The increase in pH resulted in accelerated degradation of PAsp(AE). The pH-dependent degradability of PAsp(AE) was further evaluated under varying incubation periods, and the ln*f* values were plotted against incubation time (Figure 4(a)). Then, *k* [h^–1^] and *T* [h] under each condition were calculated (Table 1). While almost no degradation occurred at pH 5.0 (*k* = 1.86 × 10^–5^), a high *k* value was observed at pH 9.0 (*k* = 2.44 × 10^–2^), over 10-fold higher than that observed at pH 7.4 (*k* = 2.11 × 10^–3^). Here, there was a possibility that the higher pH might also facilitate the degradation of side-chain amide bonds. Thus, the possibility was further verified by the same procedure as used for Figure S4. In this experiment, PAsp(AE) was incubated at pH 9.0 for 6 h to considerably degrade (Figure S9(a)). The peak intensity ratio of free EDA protons (*δ* = 3.4) to AE moiety protons (*δ* = 3.2) in the ^1^H NMR spectrum of the degraded PAsp(AE) was calculated to be ~9% (Figure S9(b)), indicating that only a slight cleavage reaction occurred in the side-chain amide bonds of PAsp(AE) at pH 9.0, similar to pH 7.4. Note that PAsp(AP) and PAsp(AB) also exhibited accelerated degradation behaviors at a higher pH, although their cleavage rate constants were much lower than that of PAsp(AE) (Table 1 and Figure S10). Ultimately, the cleavage rate constants of the PAsp(R) series were plotted against the degree of deprotonation of the primary amines (Figure 4(b)). A positive correlation between *k* and the degree of deprotonation of the primary amines was observed for the PAsp(R) series. This result indicates that the increase in deprotonated primary amines with high nucleophilicity in the side chain facilitates degradation of the polyaspartamide main chain, especially in PAsp(AE). These results strongly support our hypothesis that the interaction of the nucleophilic primary amines with the amide proton in the side chain is crucial for the degradability of the PAsp(R) series.

### 3.4. *Cytotoxicity of* N*-substituted polyaspartamides*

**Figure 5.**
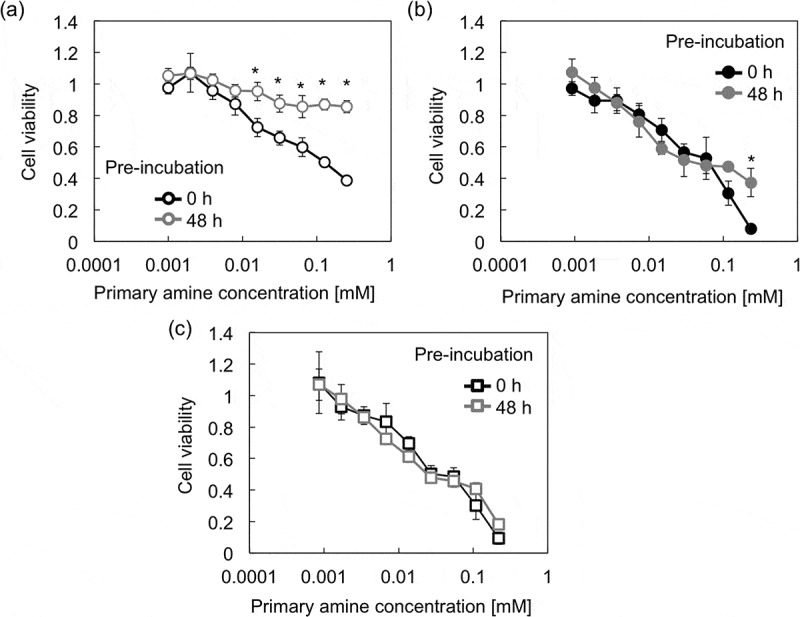
Viability of Huh-7 cells after 48 h incubation with (a) PAsp(AE), (b) PAsp(AP), and (c) PAsp(AB) at varying concentrations. Each PAsp(R) was pre-incubated for 0 or 48 h at 37 °C and pH 7.4. Results are expressed as mean ± SD (*n* = 5). The data were statistically analyzed by Student’s *t*-test in Microsoft Excel (*p < 0.0005).

It is expected that the varying degradability of the PAsp(R) series could influence their cytotoxicity, mainly because the cytotoxicity of polycations is positively correlated to the number of cations per polymer [17,28]. Thus, the cytotoxicity of PAsp(R)s was evaluated in the cultured Huh-7 cells after pre-incubation for 0 and 48 h at 37 °C and pH 7.4 (Figure 5). While almost no significant differences were observed in the cytotoxicity levels of PAsp(AP) and PAsp(AB), we observed that PAsp(AE) had significantly reduced levels of cytotoxicity after pre-incubation for 48 h. The *M*_n_ and DP of each PAsp(R) after 0 and 48 h incubation were estimated based on the *k* value, as summarized in Table S1. After pre-incubation for 48 h, PAsp(AE) was speculated to degrade into smaller fragments with a relative *M*_n_ of 0.09 and a DP of 9.5. These drastic reductions in the *M*_n_ and DP values might contribute to the significantly reduced cytotoxicity of PAsp(AE), as previously demonstrated for PAsp(DET) [17]. It should also be noted that PAsp(AP) did not alter the cytotoxicity despite the considerable degradation (relative *M*_n_ = 0.29, DP = 31), suggesting that there might be a threshold in molecular weight (or the number of cations per polymer) for low cytotoxicity. These results demonstrate that the rapid degradability of PAsp(AE) is desirable for the development of less toxic polycations.

### 3.5. Preparation of mRNA-loaded PICs and their characteristics

**Figure 6.**
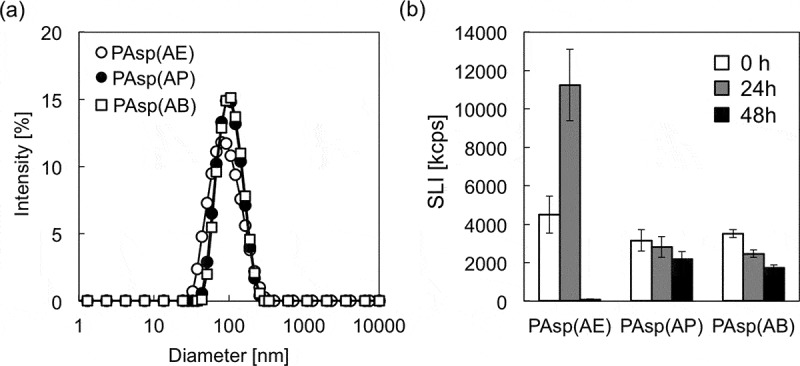
(a) Size distribution histograms of mRNA-loaded PICs prepared from PAsp(AE) (○), PAsp(AP) (●), and PAsp(AB) (□); and (b) changes in SLI of mRNA-loaded PICs after 0 h (white bar), 24 h (gray bar), and 48 h (black bar) incubation at 37 °C and pH 7.4. Results are expressed as mean ± SD (*n* = 3).

As demonstrated above, PAsp(AE) is degraded into smaller fragments at a degradation rate, e.g. *k* = 2.11 × 10^–3^ at pH 7.4. This time-dependent degradability of PAsp(AE) is expected to contribute to the drug releasability of its nanocomplex, as well as the reduced cytotoxicity. Thus, mRNA-loaded PICs were prepared as a model nanocomplex using PAsp(AE), PAsp(AP), or PAsp(AB) with *GLuc* mRNA. Note that *GLuc* mRNA was selected for following quantitative evaluation of mRNA expression efficiency based on photoluminescence intensity. All PAsp(R)s successfully formed mRNA-loaded PICs of a similar size (80–100 nm in hydrodynamic diameter) and a narrow size distribution (PDI = 0.11–0.17) (Figure 6(a) and Table S2). The SLI, size, and PDI of each mRNA-loaded PIC were further measured 24 and 48 h after incubation at pH 7.4 and 37 °C (Figure 6(b), Figure S11, and Table S2). Whereas PAsp(AP) and PAsp(AB) elicited modest decreases in SLI of their PICs with an increase in incubation time, a drastic change in SLI was observed for the PICs prepared from PAsp(AE), presumably due to destabilization of mRNA-loaded PICs associated with degradation of PAsp(AE). Particularly, the SLI of PAsp(AE) PICs appreciably increased 24 h after incubation and then decreased to the background level 48 h after incubation, suggesting disintegration of PAsp(AE) PICs 48 h after incubation.

**Figure 7.**
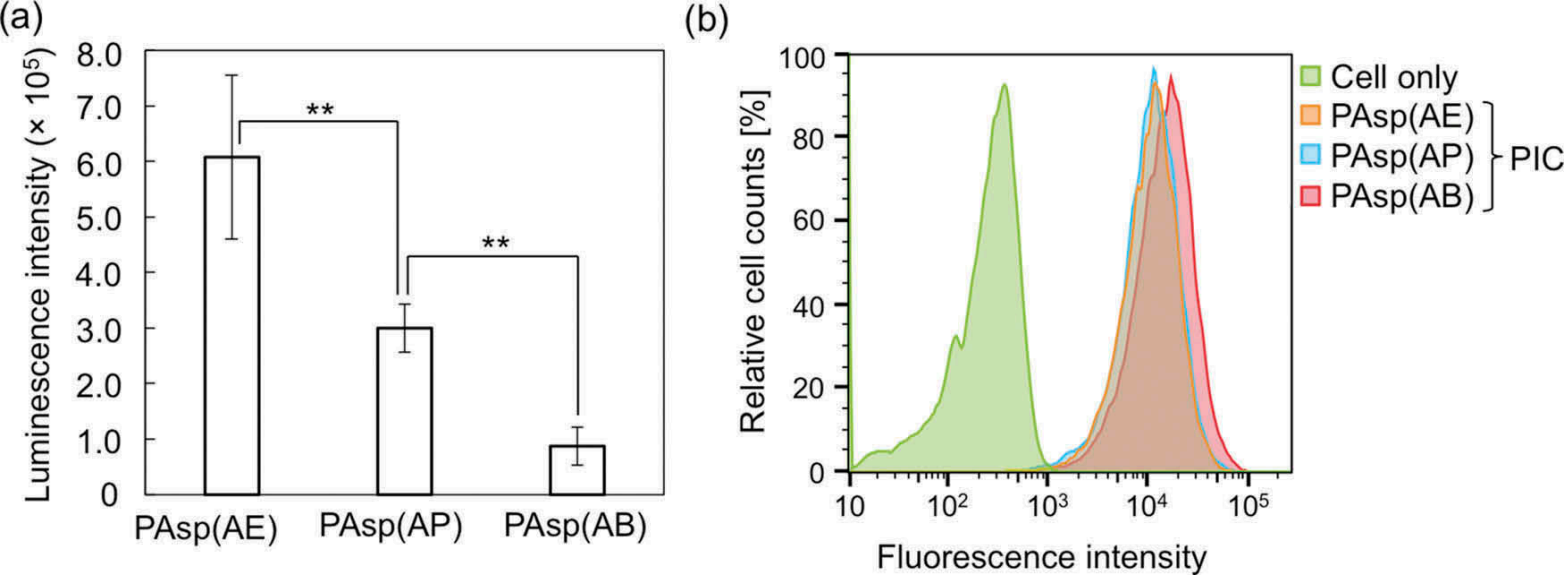
(a) *GLuc* mRNA expression level in cultured Huh-7 cells treated with *GLuc* mRNA-loaded PICs. Results are expressed as mean ± SD (*n* = 5). The data were statistically analyzed by an analysis of variance, followed by Tukey’s *post hoc* test (**p < 0.01). (b) Flow cytometry peaks showing cellular uptake of Cy5-mRNA-loaded PICs in cultured Huh-7 cells. In both experiments, 250 ng of *GLuc* mRNA was transfected to Huh-7 cells for 24 h.

Transfection efficiency of mRNA-loaded PICs was evaluated in cultured Huh-7 cells. *GLuc* mRNA-loaded PICs prepared from PAsp(AE) had a significantly higher mRNA expression, compared to those from PAsp(AP) and PAsp(AB) (Figure 7(a)). It should be noted that all tested PICs showed the similar level of low cytotoxicity under the same transfection condition (data not shown). To gain more insight into the differences in mRNA expression levels, the cellular uptake efficiency of each PIC (or Cy5-mRNA) was further evaluated by flow cytometry 24 h after transfection (Figure 7(b)). The fluorescence intensity of the internalized Cy5-mRNA in the cultured Huh-7 cells was almost similar among all the PICs, indicating that the varying mRNA expression levels were ascribed to the transfection process following the cellular internalization, e.g. endosomal escape and mRNA payload release. Here, we assumed that the endosome-escape ability of mRNA-loaded PICs should be comparable among all the PAsp(R)s because of their similar protonation states between physiological pH (7.4) and endosomal pH (~5.0) (Figure 3) and almost no degradability at the endosomal acidic pH (Figure 4(a)). Of note, the change in the protonation degree of polycations between physiological pH and endosomal pH is critical for their endosome-escape ability because the increase in the protonation degree allows for the endosome destabilization (or disruption), as extensively described in previous studies [10,13,16]. Accordingly, the higher mRNA expression by PAsp(AE) might be due to the more rapidly destabilized PICs in the cytoplasm of neutral pH (7.2) to facilitate the release of mRNA payloads for effective translation to the protein.

## 4. Conclusions

This study investigated the degradability of PAsp(R) series bearing a primary amine in the side chain. The molecular weight analyses revealed that PAsp(AE) exerted considerable degradability under physiological conditions. The degradability of PAsp(R) bearing primary amines in the side chain was significantly affected by the degree of protonation of the primary amines and the length of alkyl chain spacer in the side chain. Both the amine protonation and the longer alkyl spacer substantially suppress the degradation of the polyaspartamide main chain. Therefore, we conclude that the cleavage of the amide bonds (CONH_main_) in the main chain should be elicited by the activation (or enhanced nucleophilicity) of the amide nitrogen (CO***N***H_side_) in the side chain through abstraction (or delocalization) of the amide proton (CON***H***_side_) by the deprotonated primary amine in the side chain. In addition, the degradability of PAsp(AE) decreases the cytotoxicity and elicits destabilization of mRNA-loaded PICs, which may be advantageous for the intracellular release of mRNA allowing for enhanced mRNA expression. Indeed, the mRNA-loaded PICs prepared from PAsp(AE) had a significantly higher mRNA expression in the cultured human hepatoma cells, compared to the other derivatives with reduced degradability. Overall, our results provide a key chemical structure for fine-tuning of the nonenzymatic degradability of poly(amino acid)s, which contributes to the future design of poly(amino acid)-based biomaterials.
