# Supplementary material for: Tunable nonenzymatic degradability of N-substituted polyaspartamide main chain by amine protonation and alkyl spacer length in side chains for enhanced messenger RNA transfection efficiency
Source: Sci Technol Adv Mater. 2019 Feb 13;20(1):105–15. doi: 10.1080/14686996.2019.1569818 (PMC6374946; doi:10.1080/14686996.2019.1569818)
Supplement: Supplemental Material [file TSTA_A_1569818_SM2726.zip › suppl_data/Revised SI(Naito et al)2 for UL.docx]

**Supporting Information**

**Tunable non-enzymatic degradability of *N*-substituted polyaspartamide main chain by amine protonation and alkyl spacer length in side chains for enhanced messenger RNA transfection efficiency**

Mitsuru Naito^1^, Yuta Otsu^2^, Rimpei Kamegawa^2^, Kotaro Hayashi^3^, Satoshi Uchida^3,4^,

Hyun Jin Kim^1^, Kanjiro Miyata^2^

^1^Center for Disease Biology and Integrative Medicine, Graduate School of Medicine, The University of Tokyo, 7-3-1 Hongo, Bunkyo-ku, Tokyo 113-0033, Japan

^2^Department of Materials Engineering, Graduate School of Engineering, The University of Tokyo, 7-3-1 Hongo, Bunkyo-ku, Tokyo 113-8656, Japan

^3^Innovation Center of NanoMedicine, Kawasaki Institute of Industrial Promotion, 3-25-14 Tonomachi, Kawasaki-ku, Kawasaki 210-0821, Japan

^4^Department of Bioengineering, Graduate School of Engineering, The University of Tokyo, 7-3-1 Hongo, Bunkyo-ku, Tokyo 113-8656, Japan


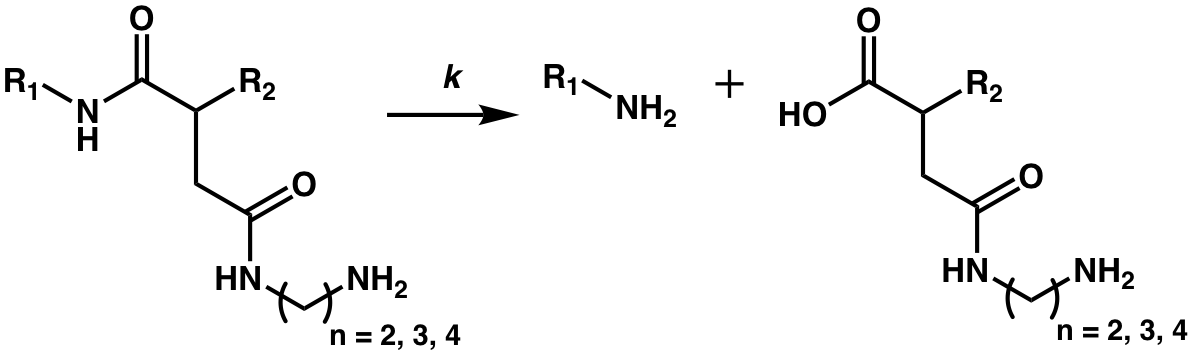


**Scheme S1**. Cleavage reaction of amide bond in PAsp(R) main chain.

**Table S1**. Estimated *M*_n_ and DP of PAsp(R)s after 0 or 48 h incubation

|  | Incubation time [h] | *M*_n_*^a,b^* | DP*^b^* |
| --- | --- | --- | --- |
| PAsp(AE) | 0 | 19,110 | 106 |
|  | 48 | 1,713 | 9.5 |
| PAsp(AP) | 0 | 20,604 | 106 |
|  | 48 | 6,019 | 31 |
| PAsp(AB) | 0 | 22,088 | 106 |
|  | 48 | 14,410 | 69 |

*^a^*Including chloride ion as a counterion; *^b^*Calculated using each *k*.

**Table S2**. Structural characteristics of mRNA-loaded PICs (mean ± S.D., n = 3)

|  | Incubation time [h]*^a^* | Size [nm]*^b^* | PDI*^b^* |
| --- | --- | --- | --- |
| PAsp(AE) | 0 h | 80 ± 4 | 0.17 ± 0.03 |
|  | 24 h | 1030 ± 249 | 0.41 ± 0.05 |
|  | 48 h | 5085 ± 1770 | 0.77 ± 0.20 |
| PAsp(AP) | 0 h | 93 ± 6 | 0.15 ± 0.02 |
|  | 24 h | 93 ± 8 | 0.18 ± 0.01 |
|  | 48 h | 81 ± 4 | 0.22 ± 0.01 |
| PAsp(AB) | 0 h | 92 ± 4 | 0.11 ± 0.01 |
|  | 24 h | 109 ± 1 | 0.15 ± 0.01 |
|  | 48 h | 101 ± 4 | 0.17 ± 0.01 |

*^a^*Incubated at pH 7.4 and 37 °C; *^b^*Determined by dynamic light scattering.


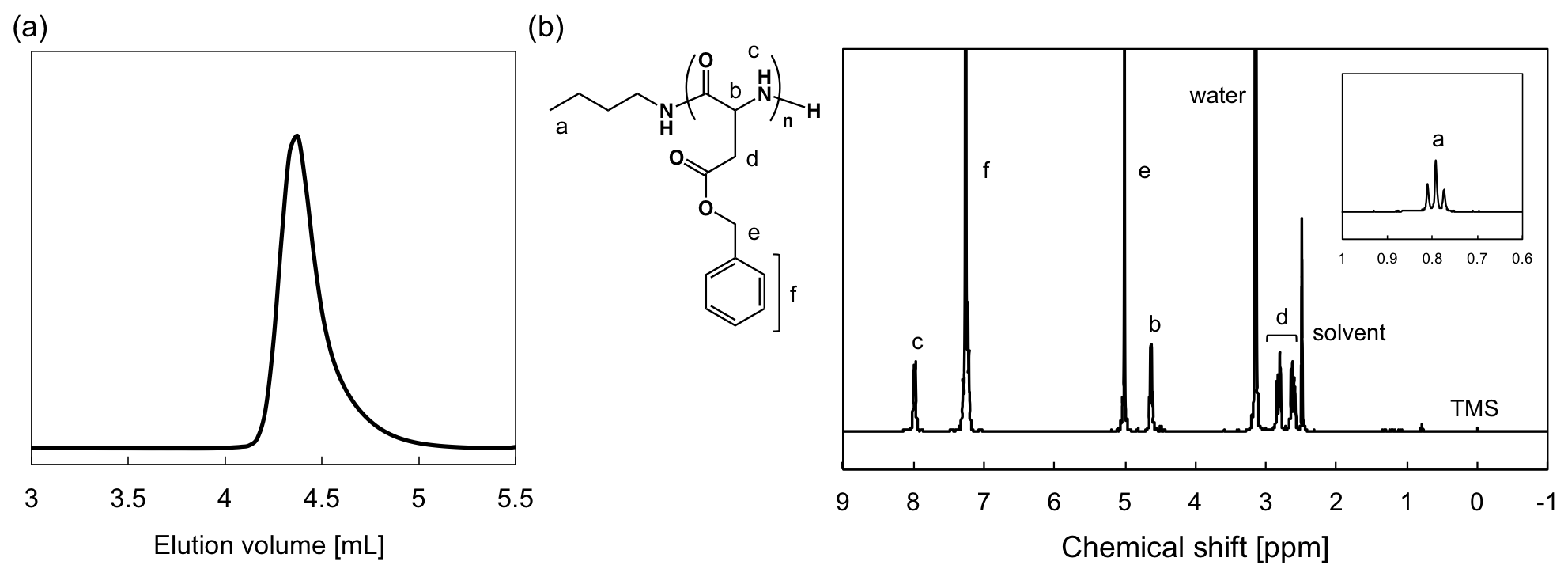


**Figure S1**. (a) SEC chart and (b) ^1^H NMR spectrum of PBLA (10 mg/mL, solvent: DMSO-*d6*, temperature: 80 °C).


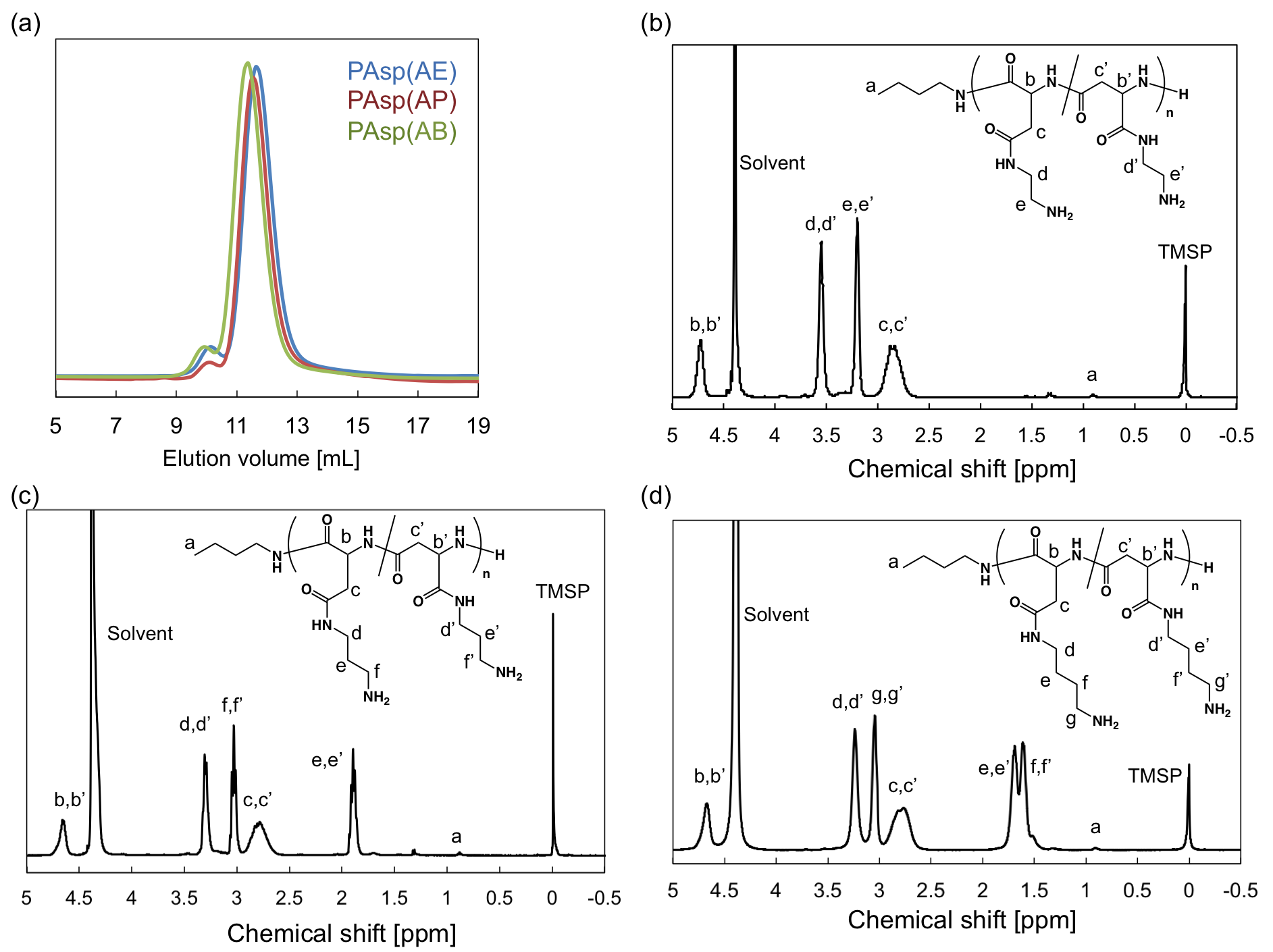


**Figure S2**. (a) SEC charts of PAsp(R) series; ^1^H NMR spectra of (b) PAsp(AE), (c) PAsp(AP), and (d) PAsp(AB) (5 mg/mL, solvent: D_2_O, temperature: 80 °C).


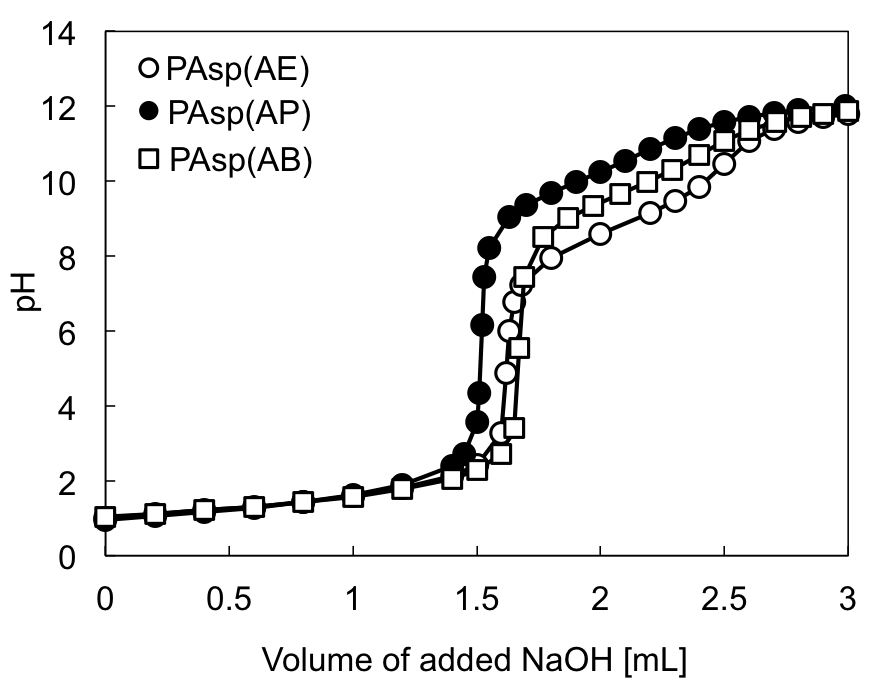


**Figure S3**. Titration curves of PAsp(AE) (○), PAsp(AP) (●), and PAsp(AB) (□) measured at 37 °C.


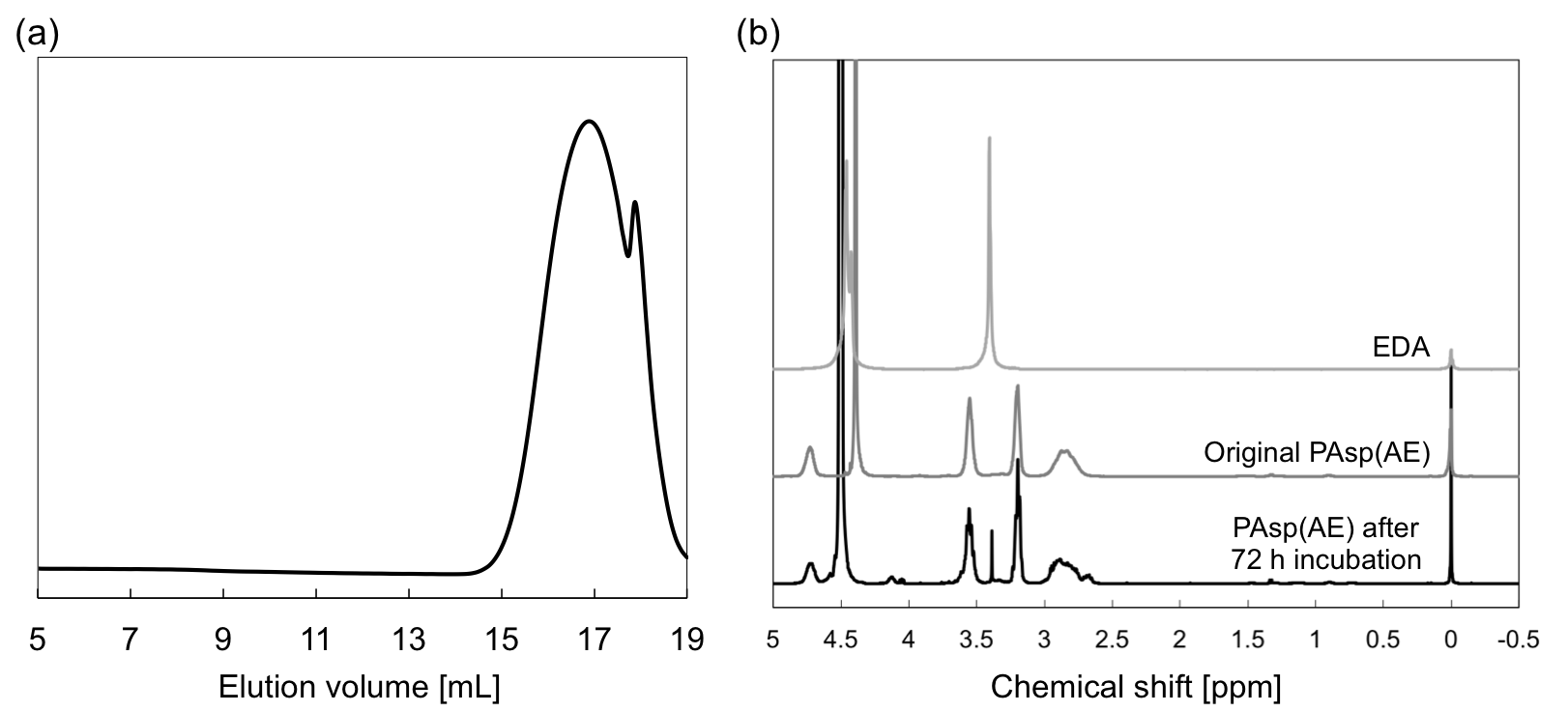


**Figure S4.** (a) SEC chart of PAsp(AE) after 72 h incubation under a physiological condition (pH 7.4, 37 °C); (b) ^1^H NMR spectra of EDA and PAsp(AE) before and after 72 h incubation (10 mg/mL, temperature: 80 °C). PAsp(AE) was incubated for 72 h in phosphate buffer (1 mg/mL, pH 7.4, 37 °C) and then lyophilized before ^1^H NMR measurement. EDA and PAsp(AE) after 72 h incubation were measured in D_2_O with 5% DCl. PAsp(AE) without incubation was measured in D_2_O.

**Figure S5**. SEC charts of (a) PAsp(AE), (b) PAsp(AP), and (c) PAsp(AB) in varying incubation times under a physiological condition (pH 7.4, 37 °C).


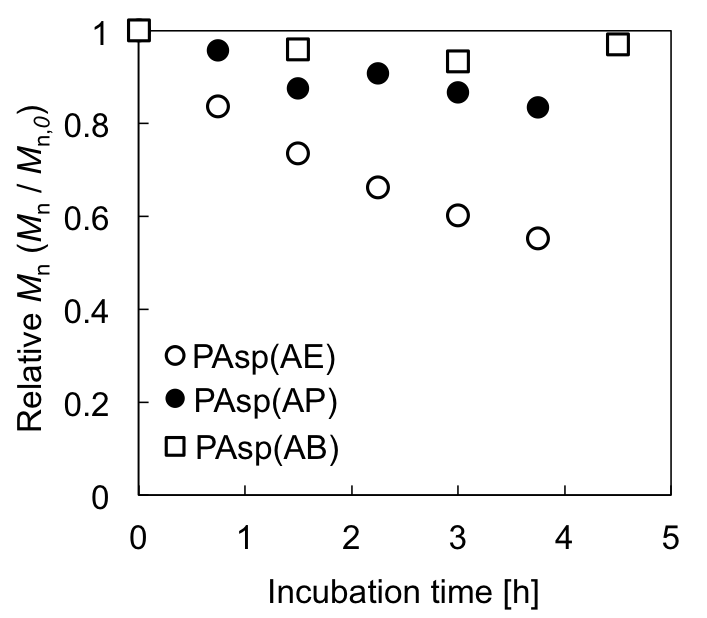


**Figure S6**. Relative *M*_n_ of PAsp(AE) (○), PAsp(AP) (●), and PAsp(AB) (□) after varying incubation times.


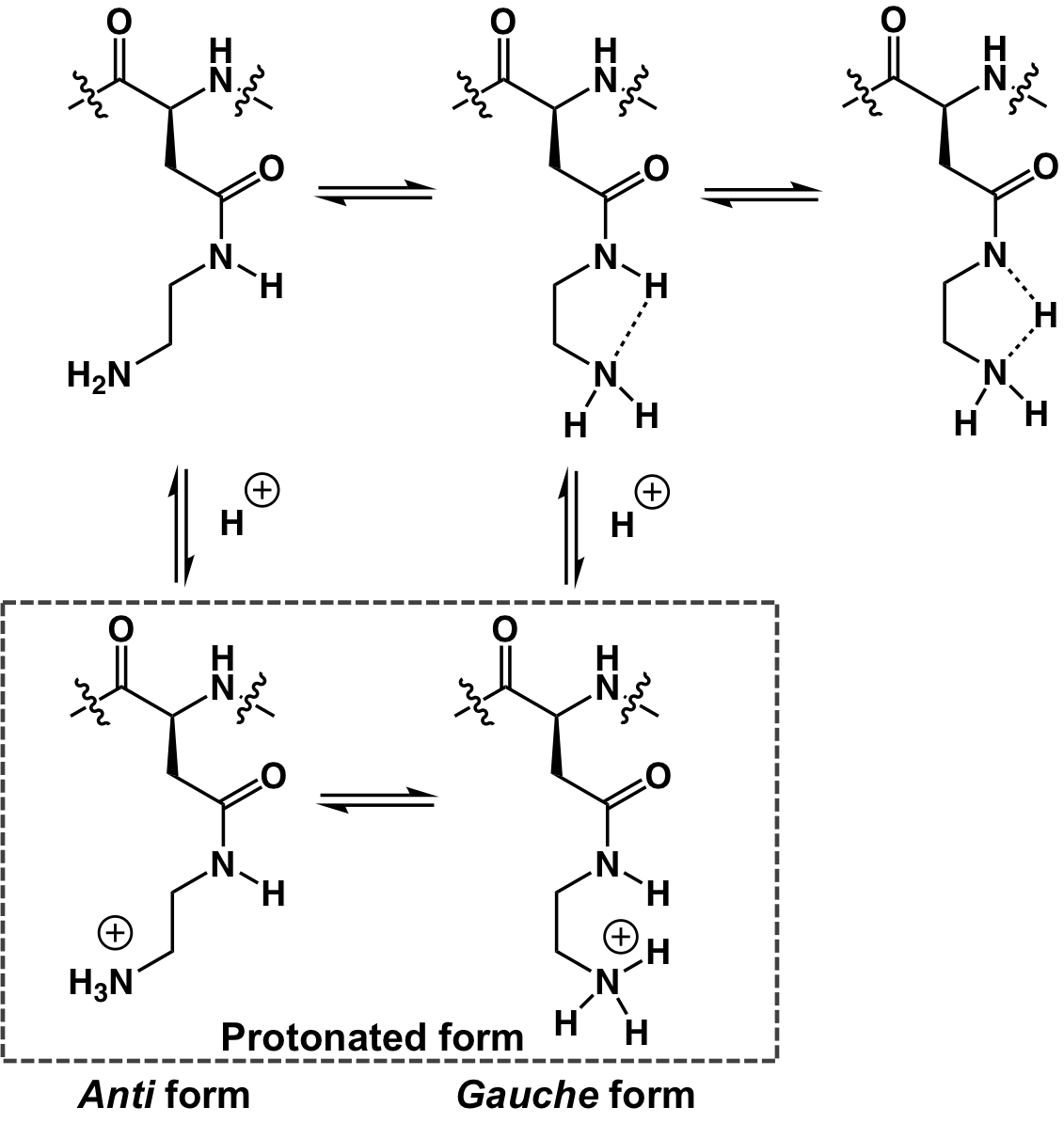


**Figure S7**. Expected conformational changes in the side chain of protonated and deprotonated Asp(AE) units.


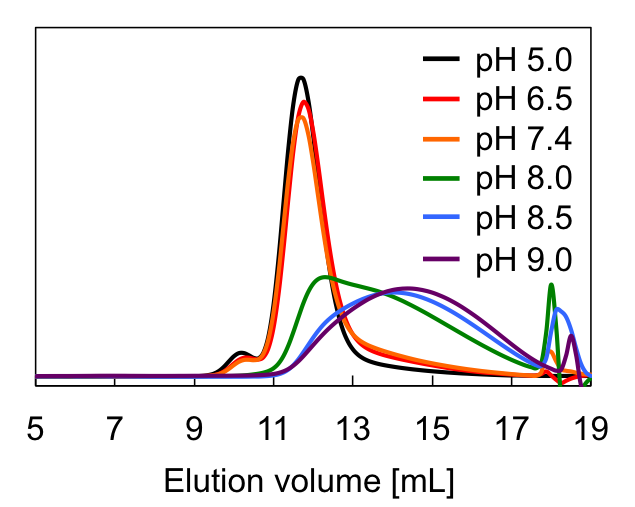


**Figure S8**. SEC charts of PAsp(AE) after 45 min incubation at different pHs.


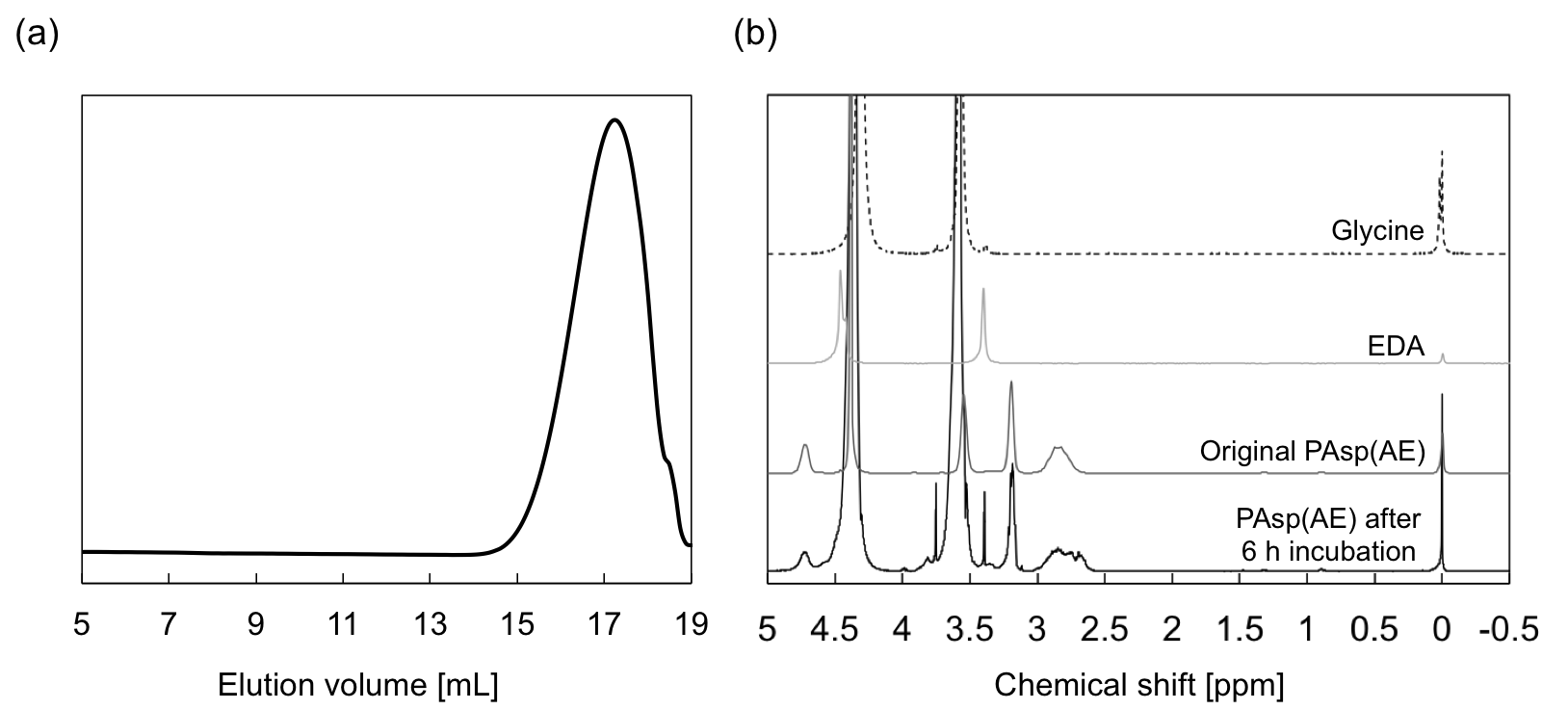


**Figure S9**. (a) SEC chart of PAsp(AE) after 6 h incubation at pH 9.0 and 37 °C; (b) ^1^H NMR spectra of glycine, EDA, and PAsp(AE) before and after 6 h incubation (10 mg/mL, temperature: 80 °C). PAsp(AE) was incubated for 6 h in glycine buffer (1 mg/mL, pH 9.0, 37 °C) and then lyophilized before ^1^H NMR measurement. Glycine, EDA, and PAsp(AE) after 6 h incubation were measured in D_2_O with 5% DCl. PAsp(AE) without incubation was measured in D_2_O.


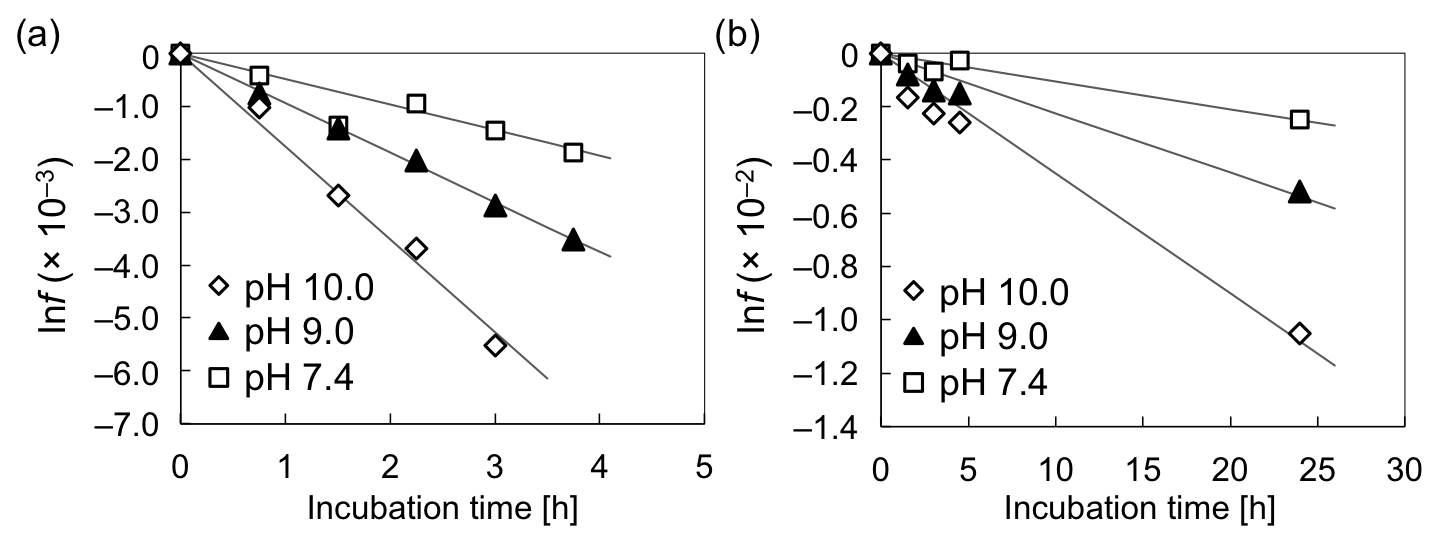


**Figure S10**. Changes in ln*f* of (a) PAsp(AP) and (b) PAsp(AB) at pH 7.4, 9.0, and 10.0 as a function of incubation time.


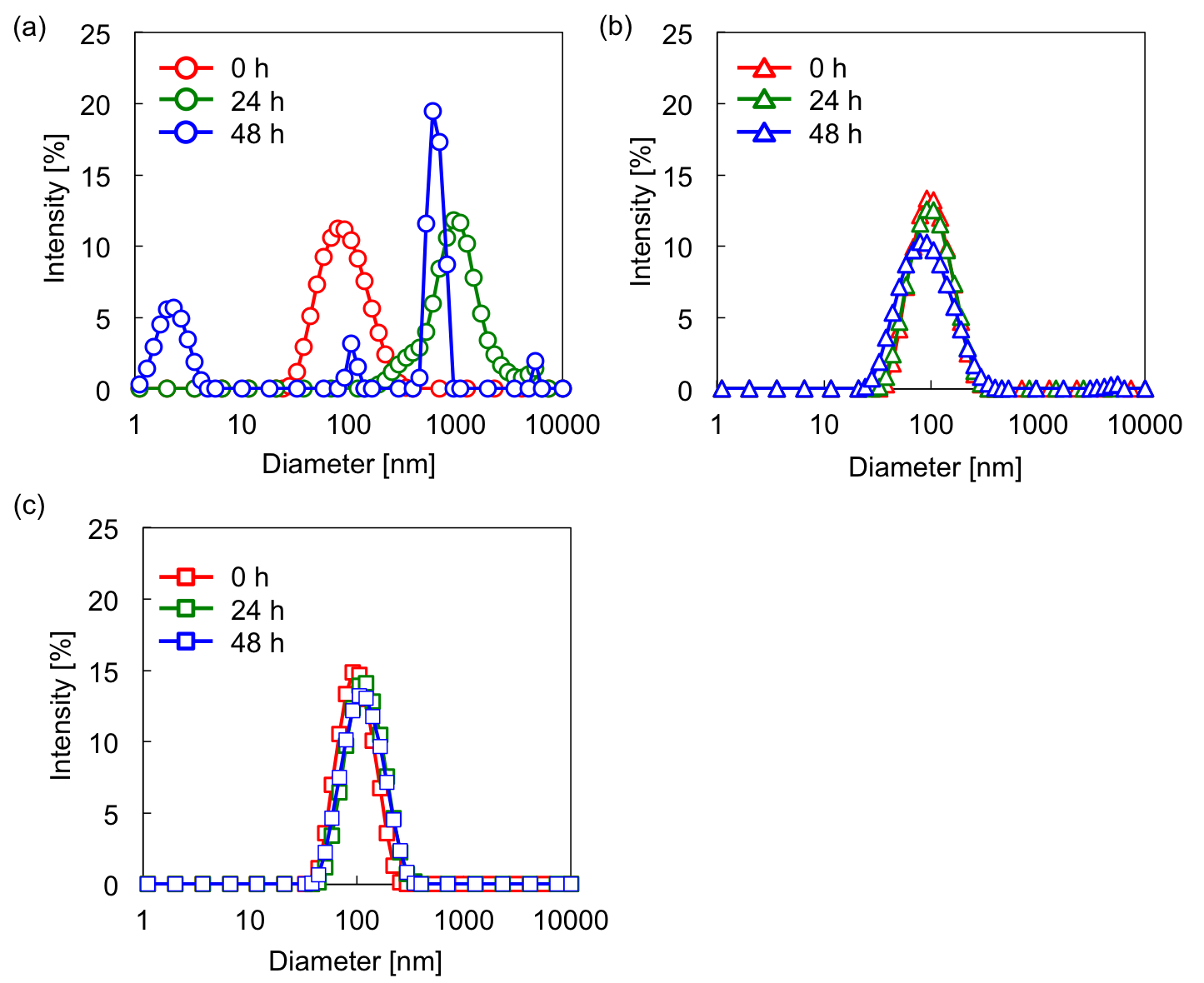


**Figure S11.** Size distribution histograms of mRNA-loaded PICs prepared from (a) PAsp(AE), (b) PAsp(AP), and (c) PAsp(AB) after 0 h, 24 h, and 48 h incubation at 37 °C and pH 7.4.
