# Supplementary figures and images for: Tunable nonenzymatic degradability of N-substituted polyaspartamide main chain by amine protonation and alkyl spacer length in side chains for enhanced messenger RNA transfection efficiency
Source: Sci Technol Adv Mater. 2019 Feb 13;20(1):105–15. doi: 10.1080/14686996.2019.1569818 (PMC6374946; doi:10.1080/14686996.2019.1569818)

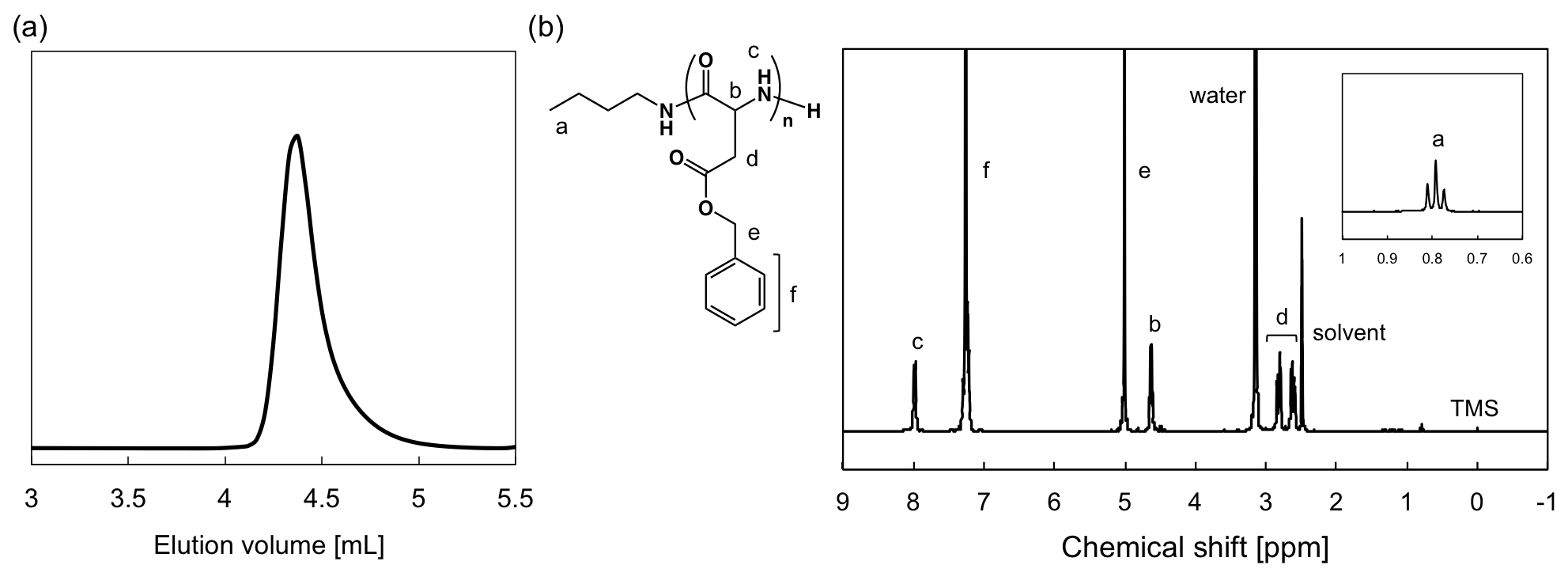

Supplement: Supplemental Material [file TSTA_A_1569818_SM2726.zip › suppl_data/Figure S1.png]

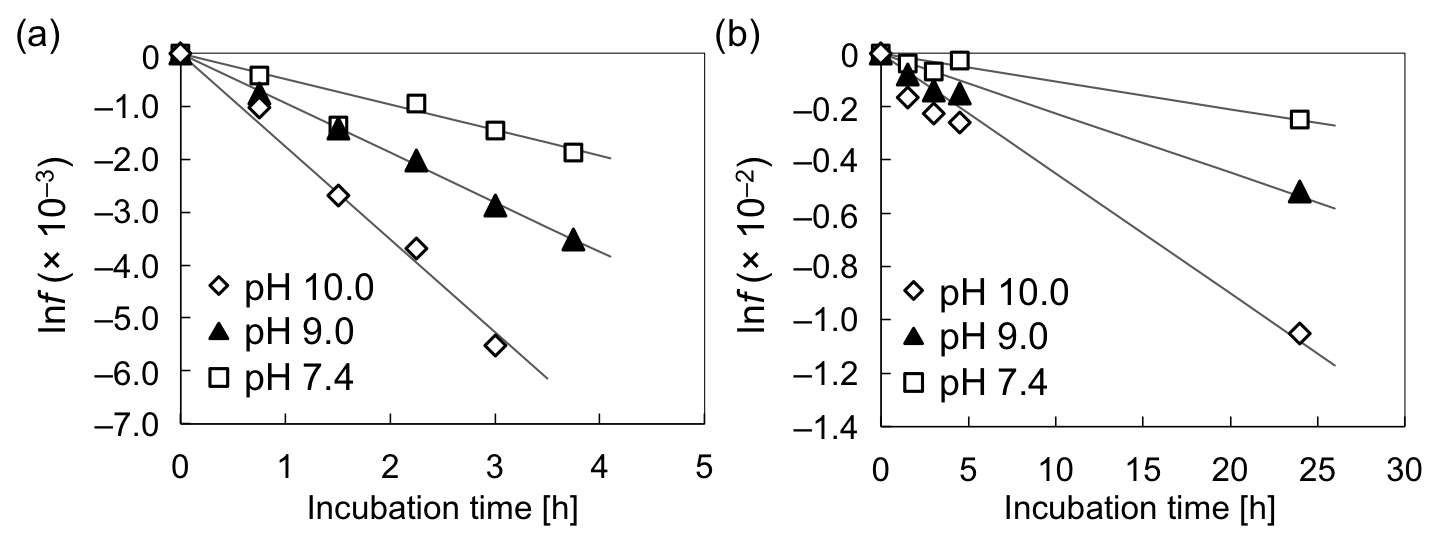

Supplement: Supplemental Material [file TSTA_A_1569818_SM2726.zip › suppl_data/Figure S10.png]

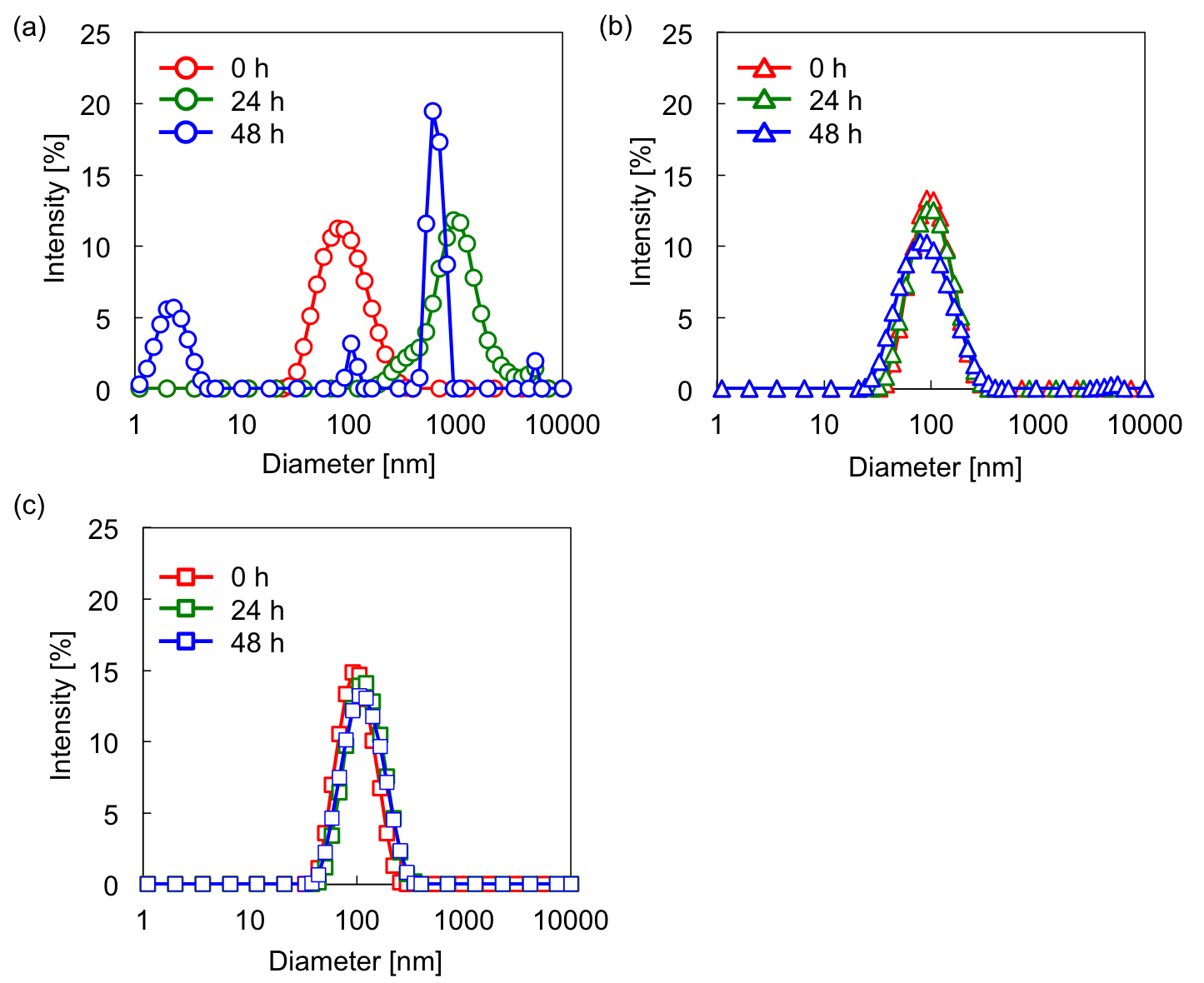

Supplement: Supplemental Material [file TSTA_A_1569818_SM2726.zip › suppl_data/Figure S11.png]

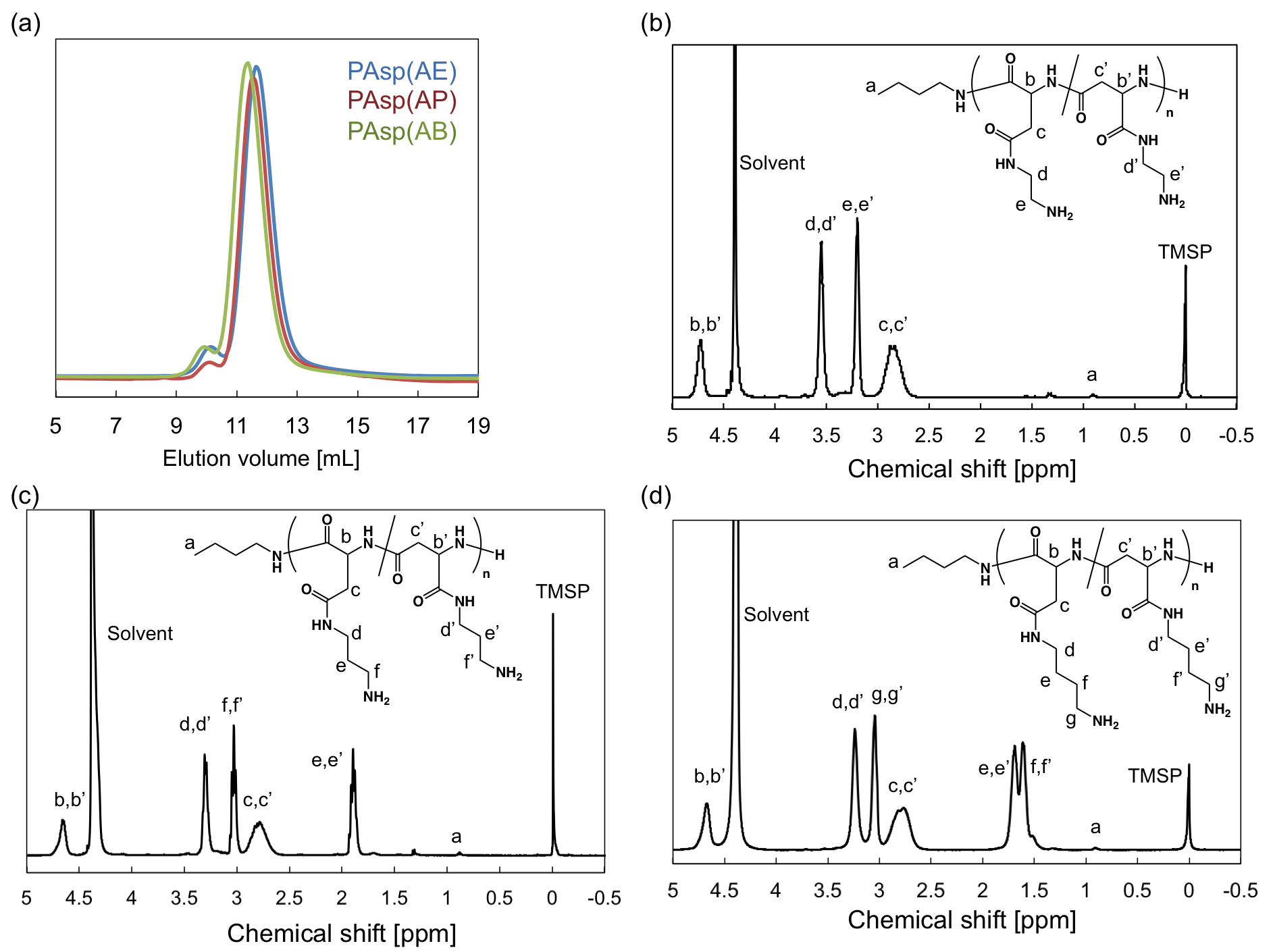

Supplement: Supplemental Material [file TSTA_A_1569818_SM2726.zip › suppl_data/Figure S2.png]

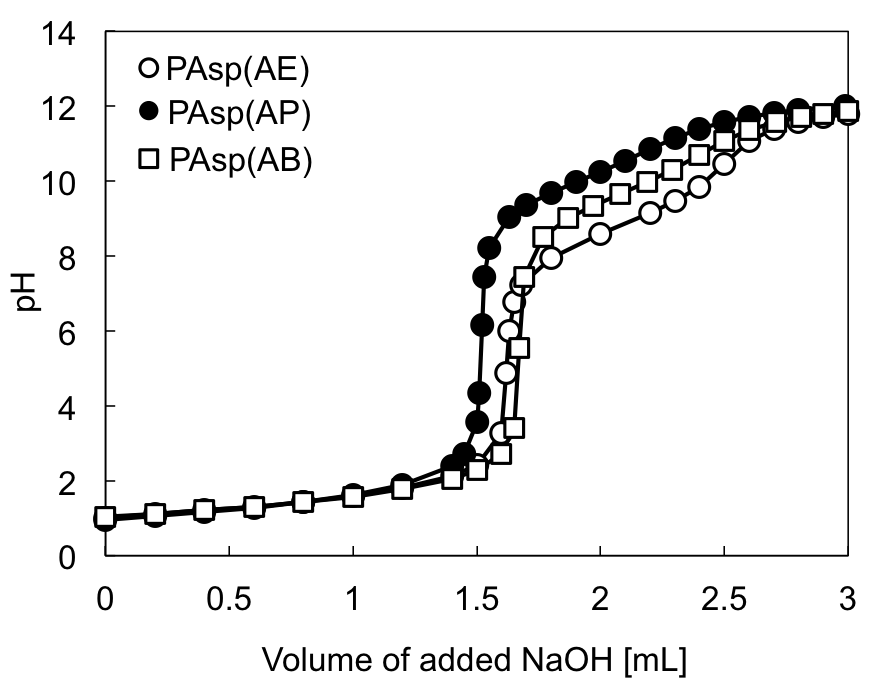

Supplement: Supplemental Material [file TSTA_A_1569818_SM2726.zip › suppl_data/Figure S3.png]

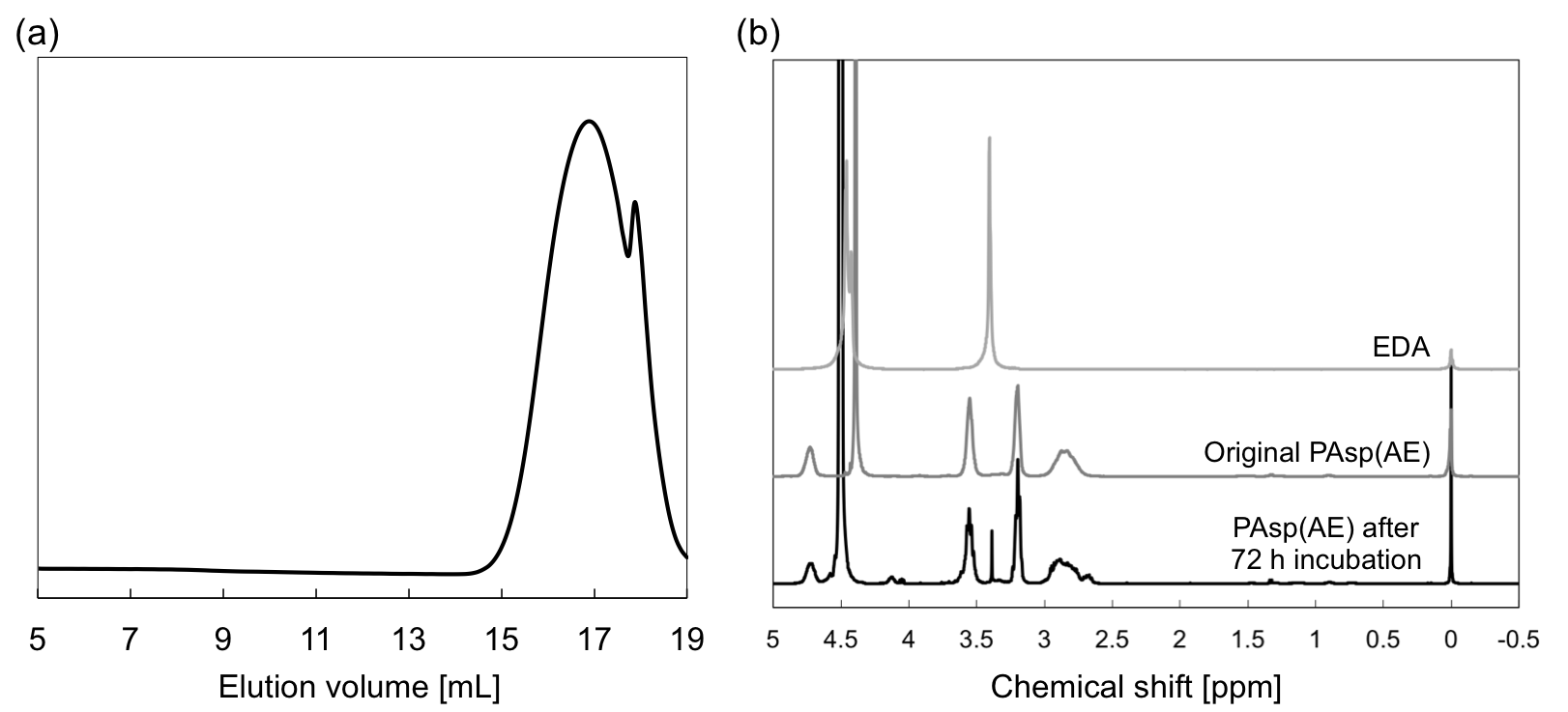

Supplement: Supplemental Material [file TSTA_A_1569818_SM2726.zip › suppl_data/Figure S4.png]

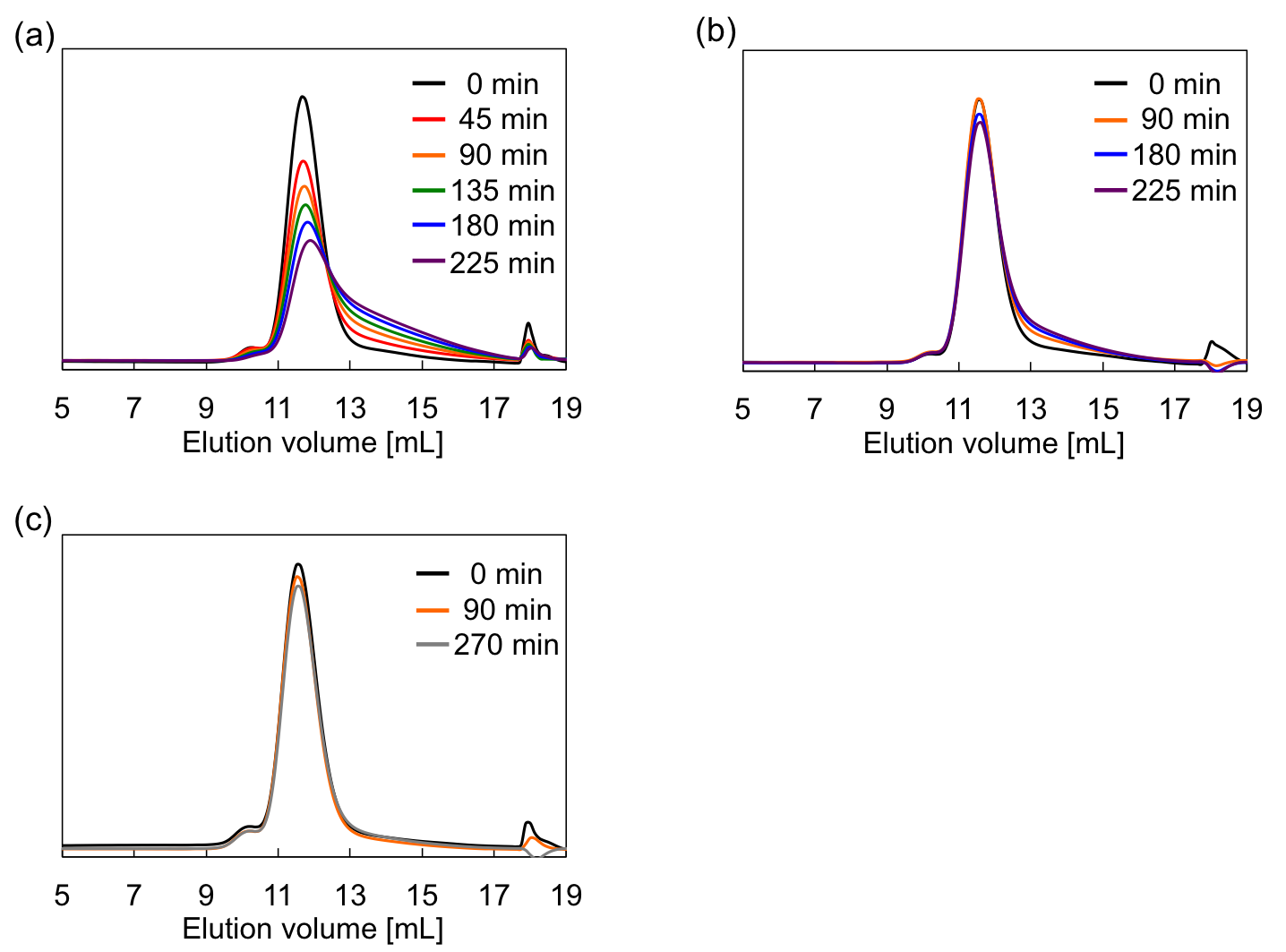

Supplement: Supplemental Material [file TSTA_A_1569818_SM2726.zip › suppl_data/Figure S5.png]

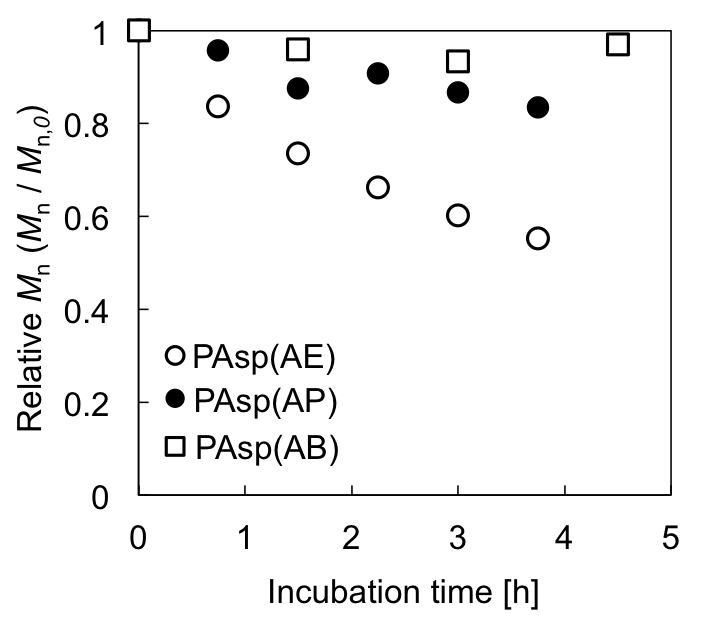

Supplement: Supplemental Material [file TSTA_A_1569818_SM2726.zip › suppl_data/FIgure S6.png]

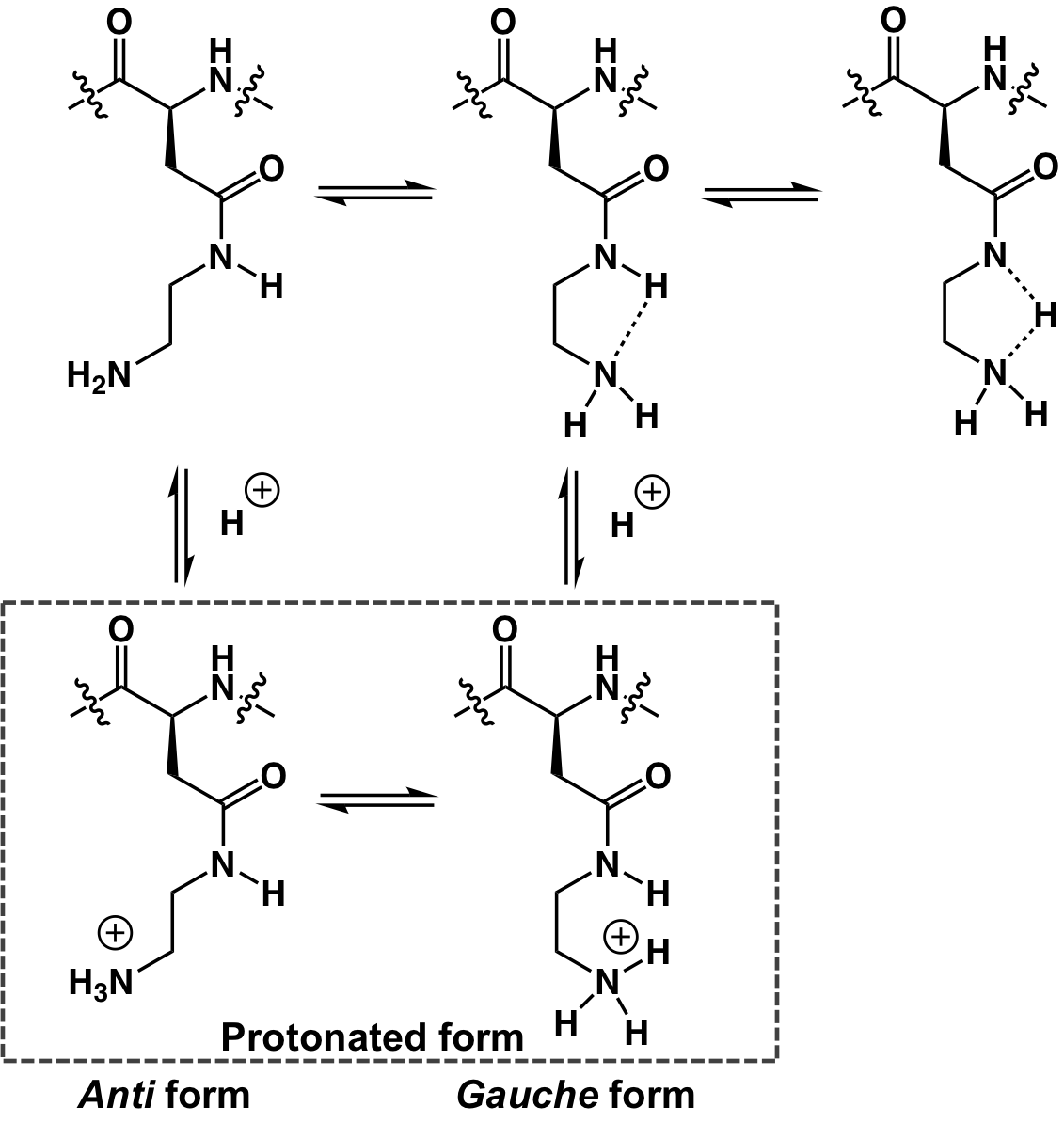

Supplement: Supplemental Material [file TSTA_A_1569818_SM2726.zip › suppl_data/Figure S7.png]

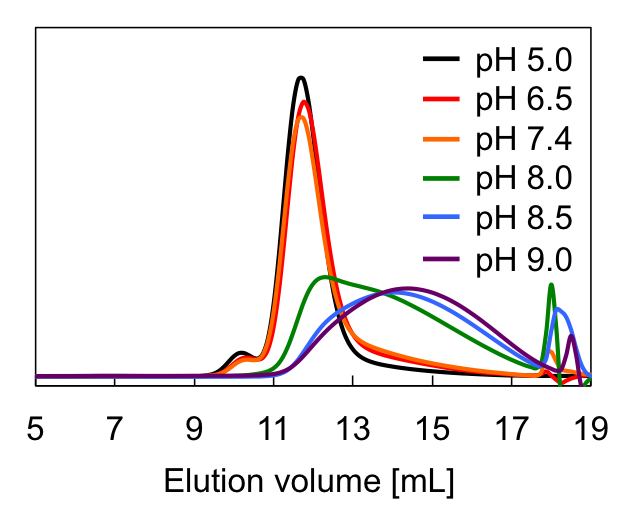

Supplement: Supplemental Material [file TSTA_A_1569818_SM2726.zip › suppl_data/Figure S8.png]

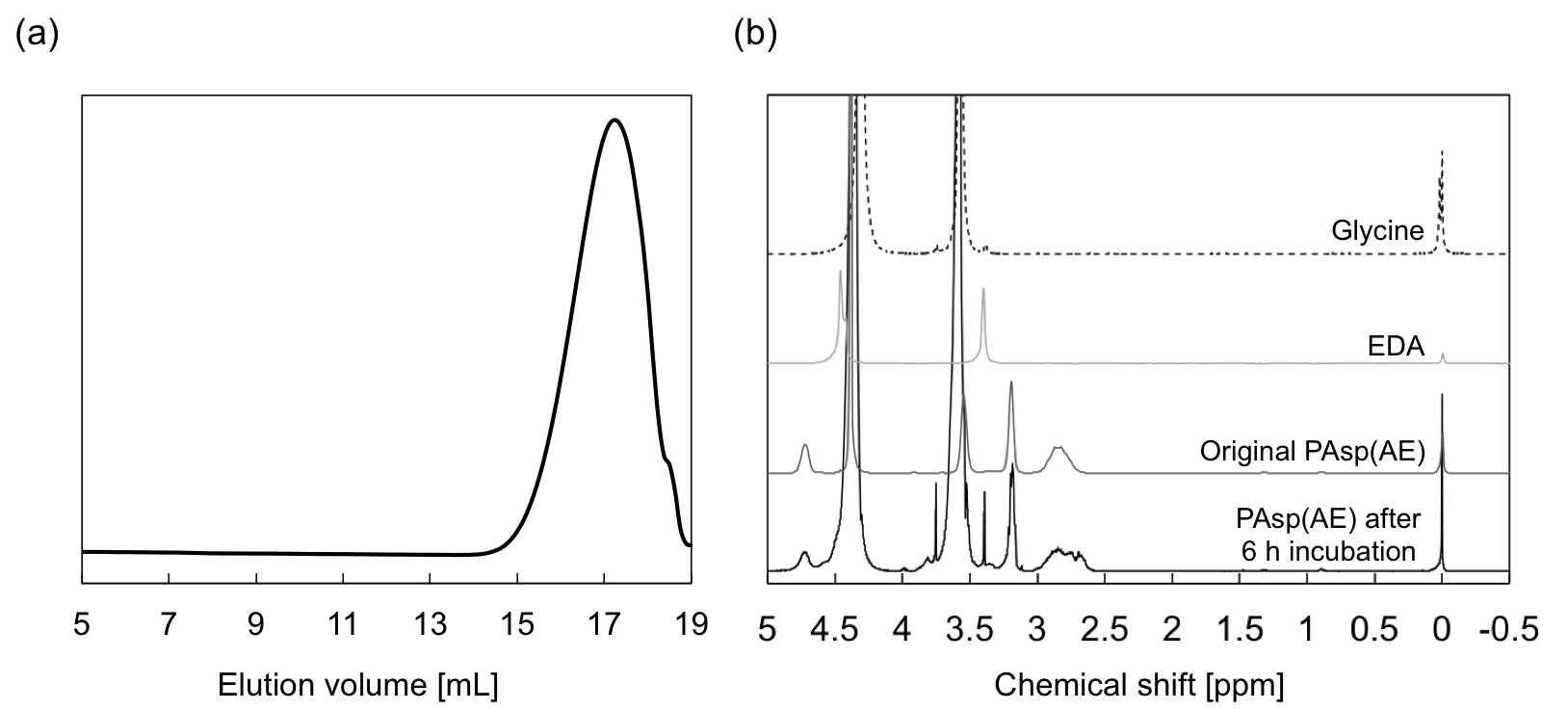

Supplement: Supplemental Material [file TSTA_A_1569818_SM2726.zip › suppl_data/Figure S9.png]

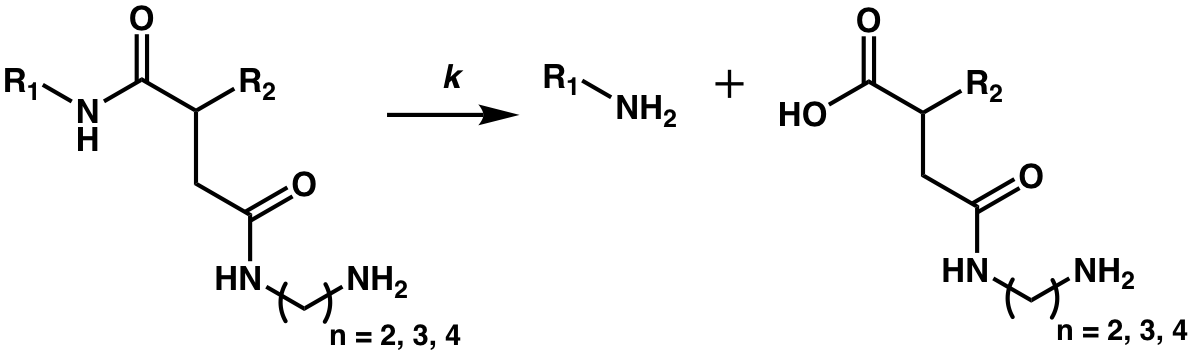

Supplement: Supplemental Material [file TSTA_A_1569818_SM2726.zip › suppl_data/Scheme S1.png]
